# A comprehensive prognostic and immune infiltration analysis of UBA1 in pan‐cancer: A computational analysis and in vitro experiments

**DOI:** 10.1111/jcmm.70037

**Published:** 2024-08-25

**Authors:** Can Chen, Yiwei Li, Zhenzhen Chen, Pengfei Shi, Yun Li, Shenxian Qian

**Affiliations:** ^1^ Department of Hematology, Affiliated Hangzhou First People's Hospital West Lake University, School of Medicine Hangzhou China; ^2^ Team of neonatal & infant development, health and nutrition, NDHN. School of Biology and Pharmaceutical Engineering Wuhan Polytechnic University Wuhan China; ^3^ Kindstar Global Precision Medicine Institute Wuhan China

**Keywords:** cancer immunity, Haematological tumours, Pan‐cancer, prognosis, *UBA1*

## Abstract

Ubiquitin like modifier activating enzyme 1 (*UBA1*) plays an important role in immune regulation and cellular function. However, the functional mechanism and role of *UBA1* in pan‐cancer have not been fully elucidated and its value in haematological tumours (diffuse large B cell lymphoma (DLBC/DLBCL) and acute myeloid leukaemia (AML/LAML)) has not been explored. We conducted a comprehensive analysis of the functional mechanism and role of *UBA1* in pan‐cancer using multiple databases, including differential expression analysis, clinical pathological staging analysis, prognosis analysis and immune analysis. Then, we confirmed the function of *UBA1* in haematological tumours through cell experiments. The results showed that the expression of *UBA1* was significantly increased in most cancers and the differential expression of *UBA1* was mainly concentrated in digestive tumours, haematological tumours and brain tumours. Moreover, the high expression of *UBA1* had poor prognosis in most tumours, which may be related to its involvement in various cancer‐related pathways such as cell cycle, as well as its methylation level, protein phosphorylation level, immune cell infiltration and immune therapy response. Cell experiments have confirmed that *UBA1* can significantly regulate the cycle progression and apoptosis of DLBCL cells and AML cells. Therefore, *UBA1* may be a potential therapeutic target for haematological tumours. In summary, our study not only comprehensively analysed the functional mechanisms and clinical value of *UBA1* in pan‐cancer, but also validated for the first time the regulatory role of *UBA1* in haematological tumours.

## 1 INTRODUCTION

Vacuoles, E1 ubiquitin‐activating enzyme, X‐linked, autoinflammatory somatic (VEXAS) syndrome is a newly discovered disease caused by somatic mutations in the Ubiquitin like modifier activating enzyme 1 (*UBA1*) gene, leading to refractory autoimmune features, often accompanied by cellular reduction.^1^ In December 2020, researchers from the National Institutes of Health in the United States first described this type of disease.^2^ The current prevalence of VEXAS syndrome is unknown and mostly occurs in males, with a median onset age of 64 years old, patients with refractory inflammatory diseases accompanied by progressive haematological abnormalities should consider the diagnosis of VEXAS.^3^ However, the prevalence of this syndrome is currently unclear. Fever, inflammation and vacuoles in haematopoietic cells are the main characteristics associated with VEXAS syndrome, VEXAS is a novel autoimmune disease prototype, characterized by somatic mutations in the *UBA1* gene encoding the enzyme 1 (E1) activator required for ubiquitin signalling, VEXAS syndrome patients exhibit a systemic autoimmune syndrome associated with haematological damage, particularly cell loss, whose pathophysiology has yet to be elucidated.^4^ VEXAS syndrome was initially diagnosed in elderly male patients and is usually associated with the diagnosis of myelodysplastic syndrome (MDS), which led the medical community to first consider VEXAS syndrome as a new subtype of MDS.^5^ However, since the first description of VEXAS patients in 2021, it seems from numerous case reports that MDS related to VEXAS is different from the MDS described in the classic, it often coexists with MDS, mainly male patients who exhibit unique clinical features of systemic inflammation after the fifth decade of life, as well as haematological abnormalities and precursor cell vacuolization in bone marrow pathology, therefore, genetic changes in the *UBA1* gene are now considered to be potentially associated with haematological tumours.^6^

Among the eight identified E1 enzymes to date, the enzyme that initiates *UBA1*, aminoacylation and nadylation is the most characteristic enzyme and is associated with various aspects of cancer biology.^7^ So far, it has been reported that more than 40 inhibitors have targeted *UBA1*, aminoacylation and nadylation, including the nadylation inhibitor Pevonilistat, which has been evaluated in more than 30 clinical trials, the clinical success of inhibitors in cancer treatment and the emergence of resistance to these drugs have prompted exploration of other signalling nodes in the ubiquitin proteasome system, including E1 enzymes.^8^ Therefore, understanding the biological characteristics of different E1 enzymes and their roles in cancer, and how to translate this knowledge into new treatment strategies has potential significance for cancer treatment.

Differences in levels of *UBA1* and Ubiquitin like modifier activating enzyme 7 (*UBA7*) were detected between squamous cell carcinoma and corresponding normal lung tissue (*p* = 0.02 and *p* = 0.01).^9^ A study has found that leukaemia cell lines and primary acute myeloid leukaemia (AML/LAML) samples have an increased dependence on *UBA1* and a decreased enzyme reserve capacity, indicating that inhibiting *UBA1* may be valuable in the treatment of AML.^10^ Some studies have revealed the sensitivity of triple negative breast cancer (TNBC) to *UBA1* depletion through genome wide CRISPR/Cas9 screening of TNBC models, targeting *UBA1* with the first type of *UBA1* inhibitor TAK‐243 induces insoluble endoplasmic reticulum stress and induces cell death by activating upregulation of transcription factor 4.^11^ Therefore, *UBA1* plays an important role in solid tumours and haematological tumours, but the specific mechanism is not clear.

Previous studies have demonstrated a potential biological association between *UBA1* and cancer.^12^ Therefore, *UBA1* may play a crucial role in the occurrence, development, and immunity of cancer. However, the biological association mechanism of *UBA1* in cancer is currently unclear. In this study, we systematically analysed the expression patterns, immune mechanisms and pathological differences of *UBA1* in pan‐cancer.

## 2 MATERIALS AND METHODS

### 2.1 Data collection and preprocessing

RNA‐sequencing expression profiles and corresponding clinical information for pan‐cancer were downloaded from The Cancer Genome Atlas (TCGA) database (https://portal.gdc.cancer.gov), Genotype Tissue Expression (GTEx) database(https://gtexportal.org/home/) and Gene Expression Omnibus (GEO) database(https://www.ncbi.nlm.nih.gov/geo/), as follows: TCGA database: 33 types of cancer, GTEx database has catalogued gene expression in >9000 samples across 53 tissues from 544 healthy individuals.^13^ GEO database: Single cell dataset of breast invasive carcinoma (BRCA) (GSE114727 and GSE148673), diffuse large B cell lymphoma (DLBC/DLBCL) (GSE175510), oesophageal carcinoma (ESCA) (GSE154763), Glioma (GSE102130, GSE131926, GSE70630 and GSE84465), liver hepatocellular carcinoma (LIHC) (GSE146115 and GSE98638), ovarian serous cystadenocarcinoma (OV) (GSE118828 and GSE154763), pancreatic adenocarcinoma (PAAD) (GSE111672, GSE148673 and GSE165399) and stomach adenocarcinoma (STAD) (GSE134520).^14^ The data from TCGA database and GTEx database were merged and subjected to batch processing effect checks, the ComBat () function in the SVA package of R software was used to remove batch processing effects. For the dataset from GEO database, the raw data was downloaded in the form of MINIML files, which contain data from all platforms, samples and geographic series (GSE) records of GSE. The extracted data was normalized through log2 transformation. The microarray data were normalized by the normalize quantiles function of the preprocessCore package in R software (version 4.2.1). Probes were converted to gene symbols according to the annotation information of the normalized data in the platform. Probes matching multiple genes were removed out from these datasets, The average expression value of gene measured by multiple probes was calculated as the final expression value. As in the case of the same dataset and platform but in different batches, used the removeBatchEffect function of the limma package in the R software to remove batch effects. After there was no batch effect in the data, we conducted subsequent differential analysis.^15^, ^16^

### 2.2 Differential expression of UBA1 in pan‐cancer

To confirm that *UBA1* was a potential oncogene, we analysed *UBA1*'s pan‐cancer expression level through the TCGA database.^17^ Gene Expression Profiling Interaction Analysis (GEPIA, http://gepia.cancer‐pku.cn/index/html) can be used to evaluate the RNA that expression data of 8587 normal TCGA samples and 9736 tumour samples, as well as the GTEx database. When obtaining *UBA1*'s expression profile, LIMMA method used for comparison between cancer group and control group, | log2FoldChange (FC) | cutof = 0.49, LogScale = log2 (TPM + 1) and q‐value cutof = 0.01 were considered to have significant differences.^18^ Based on the Human Protein Atlas (HPA) database(https://www.proteinatlas.org/), the subcellular localization of *UBA1* was obtained through immunofluorescence localization of nuclei, microtubules and Endoplasmic reticulum (ER) in A‐431 and U‐251 MG cells, the expression level and distribution of *UBA1* in the brain were analysed.^19^

### 2.3 Pan‐cancer analysis of the correlation between UBA1 expression and clinical stages

Based on the University of ALabama at Birmingham CANcer (UALCAN) web portal, the ‘Expression’ module was used to analyse the correlation between *UBA1* expression levels and pan‐cancer clinical staging in the TCGA database. Welch's T‐test estimated the significance of expression level differences between tumour subgroups based on clinical pathological characteristics (*p* < 0.05 was considered to have statistical significance).^20^

### 2.4 Pan‐cancer analysis of the prognostic value of UBA1

The ‘Survival Map’ module from the GEPIA2 online tool was used to select all TCGA malignancies in which *UBA1* expression significantly correlated with overall survival (OS). For these cancer types, according to the median expression of *UBA1*, the samples were divided into high‐expression or low‐expression subgroups for subsequent Kaplan Meier survival analysis, logrank‐p < 0.05 was considered to have significant differences.^21^ Furthermore, a univariate regression analysis (survival package of R software) was performed to evaluate the effect of *UBA1* expression on disease‐free survival (DFS), OS, disease‐specific survival (DSS) and progression‐free survival (PFS) of patients in the pan‐cancer cohort. Hazard Ratio (HR) >1 indicates that high expression of *UBA1* is a risk factor for cancer, HR <1 indicates that high expression of *UBA1* was a protective factor for cancer (*p* <0.05 was considered to have significant differences).^22^

### 2.5 UBA1 co‐expressed gene/protein interaction network and enrichment analysis

Analyse the top 100 *UBA1* co‐expressed genes in pan‐cancer on GEPIA2.0 (‘Similar Genes Detection’ module) and select the top 5 genes for correlation analysis. The correlation results are presented using a heatmap, red represents positive correlation, blue represents negative correlation, *p* < 0.05 represents significant correlation. Then further investigate the potential impact of *UBA1* on protein interaction networks. A Protein–Protein Interaction Networks (PPI) network that was centered on *UBA1* was constructed by GeneMANIA (https://genemania.org/), which included the correlation data of genetic interactions and protein, pathways, co‐expression, co‐localization and protein domain similarity. Then gene ontology (GO) function enrichment and Kyoto Encyclopedia of Genes (KEGG) pathway analysis were carried out for the gene that was centered on *UBA1* that was constructed by GeneMANIA. *p* < 0.05 identified as significant.^23^

### 2.6 Function and pathway enrichment analysis

Metascape (https://metascape.org/gp/index.html#/main/step1) was a website for analysing protein lists or gene, which was used to analyse the functional clustering of gene sets. ClusterProfiler package was used to analyse the gene set of KEGG and GO, *p* < 0.05 is considered significant.^24^ Conduct gene set enrichment analysis (GSEA) to study the biological signal pathway between low and high Hub gene expression. *p* < 0.05 was considered significant.^25^

### 2.7 Pan‐cancer analysis of methylation levels and UBA1 mutations

The methylation levels of the *UBA1* promoter were compared between pan‐cancer and relevant normal tissues by querying the ‘methylation’ module of the UALCAN web portal. The promoter methylation level was expressed by β‐values, with 0.25–0.3 being considered hypermethylation and 0.5–0.7 being considered hypomethylation, *p* < 0.05 is considered significant.^26^, ^27^ Information on *UBA1* genetic alteration features of all pan‐cancer samples was queried by logging into the cBioPortal database (http://www.cbioportal.org/), such as mutation frequencies, alteration types, copy number alteration (CNA) and the potential relationship between *UBA1* gene mutations and prognosis (OS) in patients with different types of cancer. logrank Test *p*‐Value <0.05 was considered significant.^28^

### 2.8 Pan‐cancer analysis of UBA1 phosphorylation

The protein phosphorylation data of Clinical Proteomic Tumour Analysis Consortium (CPTAC) samples in the UALCAN database were adopted to compare phosphorylation levels of *UBA1* protein between tumour and matched paracancerous tissues, the ‘PhosphoProtein’ module was used to perform this analysis. *p* < 0.05 was considered significant.^29^

### 2.9 Pan‐cancer analysis of the correlation between UBA1 expression and immune regulatory factors tumour mutational burden (TMB), microsatellite instability (MSI), and mismatch repair (MMR) related genes

The Spearman correlation analysis (corrplot package of R software) was performed to show the associations between *UBA1* and reported biomarkers of cancer immunotherapy for each cancer type. The relationship between *UBA1* and TMB and MSI was also analysed in pan‐cancer by Spearman correlation analysis.^30^ In tumours, MMR functional defects are usually caused by pathogenic mutations in the MMR gene(*MLH1*, *MSH2*, *MSH6* and *PMS2*)and related gene *EPCAM*. *EPCAM* was widely expressed in various malignant tumours, especially in digestive system tumour cells. *EPCAM* was not an MMR gene, but it was an upstream gene of *MSH2*, *EPCAM* deficiency can lead to epigenetic inactivation of *MSH2*, causing *MSH2* silencing and disrupting the MMR pathway. Therefore, in order to investigate whether *UBA1* expression can predict tumour progression, we selected these five genes and evaluated their relationship with *UBA1*. *p* < 0.05 was considered significant.^31^

### 2.10 UBA1 and immune infiltration, single‐cell, immune checkpoint and immune factors

Firstly, we investigated the correlation between *UBA1* expression and immune cell infiltration levels, using the XCELL method (xCell package of R software) to demonstrate the landscape of *UBA1* associated with various immune cell infiltration, Specifically, Spearman correlation analysis was conducted between immune infiltration scores and *UBA1* gene expression levels in pan‐cancer tissues and the results were presented using a heatmap. The horizontal axis represents different tumour tissues, the vertical axis represents different immune infiltration scores, different colours represent correlation coefficients, negative values represent negative correlation, positive values represent positive correlation, and the stronger the correlation, the darker the colour. *p < 0.05, **p < 0.01, ***p < 0.001 and asterisks represent importance (*p).^32^ Secondly, in order to analyse the differences in *UBA1* expression at the pan‐cancer single‐cell level, *UBA1* expression data from single‐cell subtypes were retrieved from Tumour Immune Single‐cell Hub (TISCH) (http://tisch.comp‐genomics.org/). Subsequently, we analysed the correlation between *UBA1* and 8 immune checkpoint genes using R 4.2.1, Specifically, Spearman correlation analysis was performed on the expression levels of immune checkpoint related genes and *UBA1* genes in pan‐cancer tissues. The results were presented using heatmaps, with the horizontal axis representing different immune checkpoint genes and the vertical axis representing different tumour tissues. Each box in the figure represents the correlation analysis between *UBA1* gene expression and immune checkpoint related genes expression in corresponding tumours, with *p < 0.05, **p < 0.01, ***p < 0.001, asterisks representing importance (*p), and different colours representing changes in correlation coefficients.^33^ Finally, we analysed the differential expression of *UBA1* in different immune subtypes of cancer using the tumour‐immune system interactions database (TISIDB) (http://cis.hku.hk/TISIDB/). And analysed the correlation between *UBA1* and chemokines, receptors and immunostimulants. *p* < 0.05 was considered significant.^34^

### 2.11 UBA1 predicts treatment response to pan‐cancer and drug sensitivity

In order to investigate whether *UBA1* can predict treatment response to cancer, data was obtained from ROCplotter(https://www.rocplot.org/), At present, the database only supports online analysis of these four types of cancer (BRCA, OV, glioblastoma multiforme (GBM) and colorectal cancer (CRC)).^36^ *p* < 0.05 is considered significant. The drug sensitivity analysis of *UBA1* expression in tumours was studied using GSCALite (http://bioinfo.life.hust.edu.cn/GSCA/#/). This tool was used to calculate the relationship between gene expression and drug sensitivity (50% inhibition concentration). Red indicates positive correlation. *p* < 0.05 was considered significant.^35^

### 2.12 Construction of UBA1 overexpression DLBCL cell model

OCI‐LY1 cells were inoculated in dulbecco's modified eagle medium (DMEM) that contained 10% fetal bovine serum (containing 100 mg/mL streptomycin and 100 U/mL penicillin) and cultured at 37°C in 5% CO_2_ incubator. When the adherent parietal cell grows into a compact monolayer, it is subcultured. Partial stably growing DLBCL cancer cells were divided into two groups randomly: the empty control group (OCI‐LY1 + NC group) and the *UBA1* overexpression group (OCI‐LY1 + *UBA1*‐Overexpression group). The nonsense sequence *UBA1* plasmid vector and the *UBA1* overexpression plasmid vector were transfected into OCI‐LY1 + NC group cells and OCI‐LY1 + *UBA1*‐Overexpression group cell, respectively. In addition, Quantitative Real‐time PCR (qPCR) was used to verify the overexpression effect of *UBA1* in OCI‐LY1 + NC group cells and OCI‐LY1 + *UBA1*‐Overexpression group cells. Student's t‐test (unpaired) was used to check the statistical significance while comparing the means of two groups, each group has three replicates. *p* < 0.05 was considered significant.^36^

### 2.13 Construction of UBA1 interference AML cell model

SiRNA is a chemically synthesized small molecule serving as an important intermediate for gene that silences and sequence specific RNA degradation. It had special structural features such as a 5 ‘end phosphate group and a 3’ end hydroxyl group, with two free bases at the 3 ‘end of each of its two chains. It degrades mRNA through specific complementary binding with the target mRNA. We constructed three siRNAs (*UBA1*‐siRNA1, *UBA1*‐siRNA2, and *UBA1*‐siRNA3) based on the *UBA1* sequence, a random sequence was used as the control (*UBA1*‐NC) group. After transfection into HL‐60 cells, these three siRNAs’ interference effect on *UBA1* was verified through qPCR. and *UBA1* interference's cell model was constructed by selecting one of the three siRNAs with the significant interference effect and best interference effect. Each group has three replicates, Ordinary one‐way ANOVA for multi‐group comparative analysis. *p* < 0.05 was considered significant.^37^

### 2.14 Cycle experiments of DLBCL cells and AML cells

After corresponding culture stimulation, the culture medium of DLBCL cells (OCI‐LY1 + NC group cells and OCI‐LY1 + *UBA1*‐Overexpression group cells) and AML cells (*UBA1*‐NC group cells and HL‐60 + *UBA1*‐siRNA1 group cells) were transferred to centrifuge tubes. The centrifuge tube was centrifuged at 4°C for 5 min (1000 rpm) and the supernatant was removed, 3 mL of pre‐cooled phosphate buffer saline (PBS) was added to a centrifuge tube to resuspend cells. The centrifuge tube was centrifuged at 4°C for 5 min (1000 rpm) and the supernatant was removed, pre‐cooled 75% alcohol was added to a centrifuge tube to resuspend and fix the cells and placed in a refrigerator at 4°C for overnight fixation. Subsequently, pre‐cooled PBS was added to the centrifuge tube and washed three times. The centrifuge tube was centrifuged at 4°C for 5 min (1000 rpm) before removing the supernatant. Finally, Propidine iodide (PI) staining solution was added and stained in the dark at 37°C for 30 min before flow cytometry detection. We analysed three independent repeated data and plotted them, Student's *t*‐test (unpaired) was used to check the statistical significance while comparing the means of two groups. *p* < 0.05 was considered significant.^38^

### 2.15 Apoptosis experiments of DLBCL cells and AML cells

DLBCL cells (OCI‐LY1 + NC group cells and OCI‐LY1 + *UBA1*‐Overexpression group cells) and AML cells (*UBA1*‐NC group cells and HL‐60 + *UBA1*‐siRNA1 group cells) were collected (1 × 10^6^ cells/time) and washed with pre‐cooled PBS. Then we resuspended the cells using 1 mL 1× Binding Buffer and achieved a density of 1 × 10^6^ cells/ml in the tube. Then, 5 μ L Annexin V‐FITC was added to the tube and gently mix for 10 min at room temperature and in dark conditions. Finally, 5 μ L PI was added to the tube for incubation and at room temperature and in dark conditions for 5 min and then detected by flow cytometry within 1 hour. After counting total cells and apoptotic cells, we analysed three independent repeated data and plotted them. Student's t‐test (unpaired) was used to check the statistical significance while comparing the means of two groups. *p* < 0.05 was considered significant.^39^

### 2.16 Statistical analysis

Differential gene expression's most statistical analyses were performed using on‐line databases and R 4.2.1. Student *t*‐test and Welch's *t*‐test were used for comparison between the two groups. Ordinary one‐way ANOVA for multi‐group comparative analysis. The Benjamin Hochberg method was used to correct for the significance p‐value obtained from the null hypothesis test and False Discovery Rate <0.05 was used as the screening criterion. Kaplan Meier method was used for log‐rank test and survival analysis. The Spearman analysis method is used for correlation analysis. For the analysis of gene expression differences between different groups, FC >1.4 or FC <0.7 and *p* < 0.05 were considered significant. Except for the analysis of gene expression differences between different groups. all analyses with *p* < 0.05 were considered significant.^40^, ^41^

## 3 RESULTS

### 3.1 Differential expression of UBA1 in pan‐cancer

**FIGURE 1:**
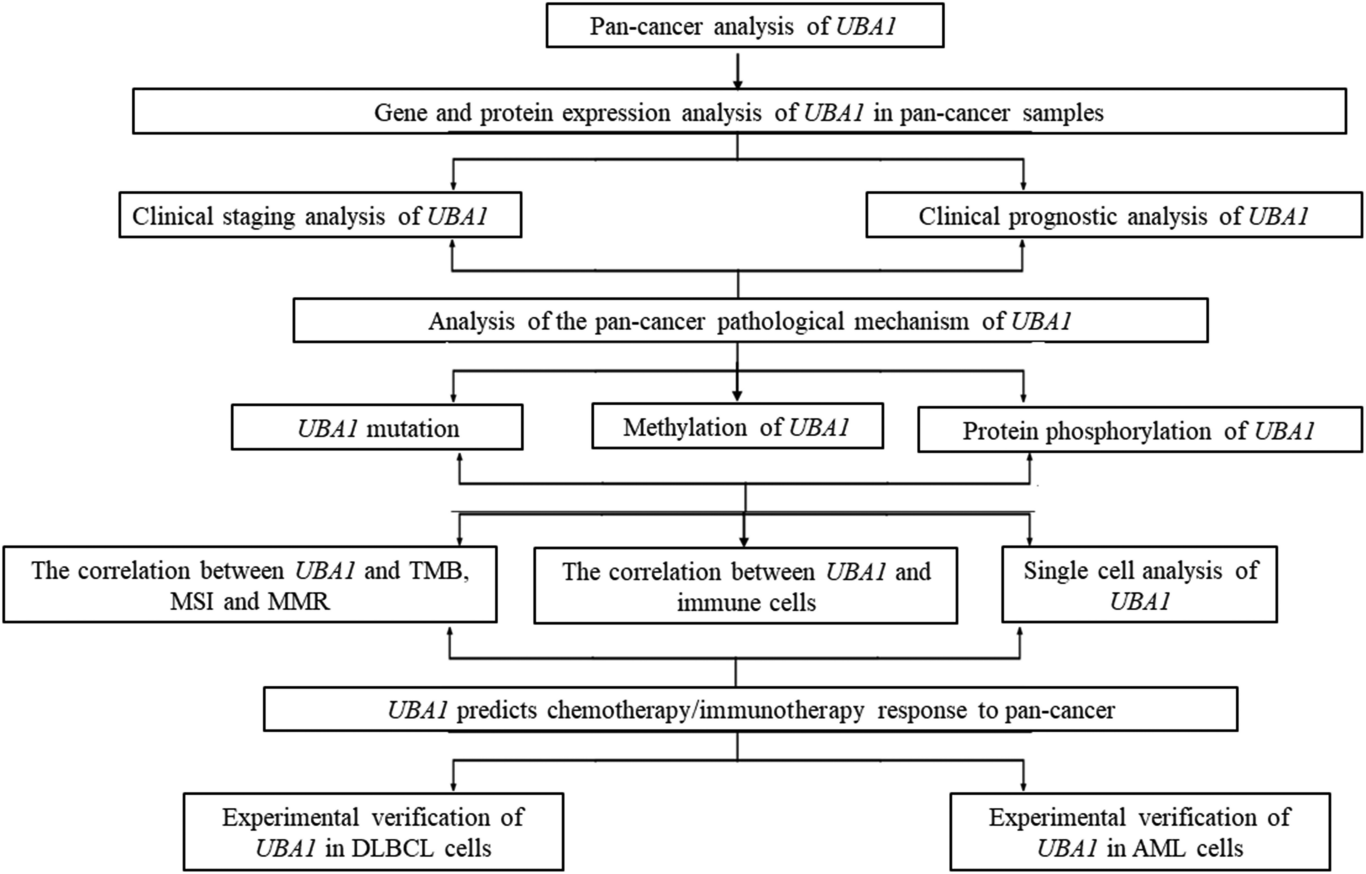
The workflow of this study.

**FIGURE 2:**
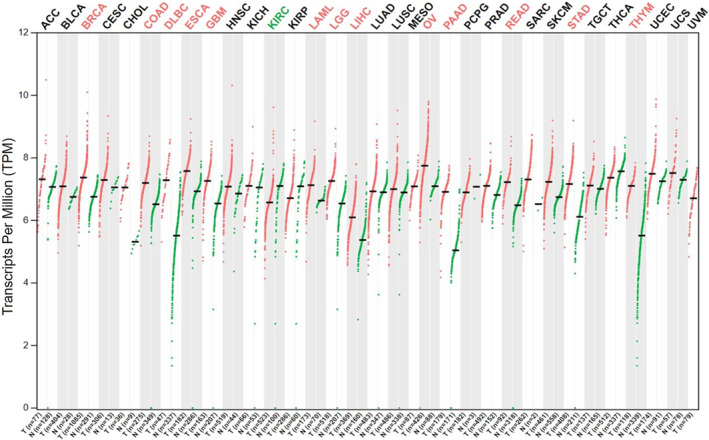
Differential expression of *UBA1* in pan‐cancer. The GEPIA2.0 results showed that *UBA1* mRNA in cancer was highly expressed in BRCA, COAD, DLBC, ESCA, GBM, LAML, LGG, LIHC, OV, PAAD, READ, STAD and THYM and significantly downregulated in KIRC (FC >1.4 or <0.7 and *p* < 0.01).

The workflow of this study is shown in Figure 1. The GEPIA2.0 results showed that *UBA1* mRNA in pan‐cancer was highly expressed in BRCA, colon adenocarcinoma (COAD), DLBC, ESCA, GBM, LAML, brain lower grade glioma (LGG), LIHC, OV, PAAD, rectum adenocarcinoma (READ), STAD and thymoma (THYM), and significantly downregulated in kidney renal clear cell carcinoma (KIRC) (FC >1.4 or <0.7 and *p* < 0.01) (Figure 2). UBA1 is significantly overexpressed in digestive tumours (COAD, ESCA, LIHC, PAAD, READ and STAD), haematological tumours (DLBCL and LAML/AML) and brain tumours (LGG and GBM). Based on the HPA database, the subcellular localization of *UBA1* was obtained through immunofluorescence localization of nuclei, microtubules and ER in A‐431 and U‐251 MG cells. *UBA1* is mainly located in Nucleopasm (Figure S1A). We further demonstrated the expression level and distribution of *UBA1* in the brain (Figure S1B–D).

### 3.2 Pan‐cancer analysis of the correlation between UBA1 expression and clinical stages

In order to investigate the relationship between the expression of *UBA1* and clinical pathological features in various cancers, we evaluated the expression of *UBA1* in stages I, II, III and IV of cancer patients. The results from the TCGA database showed that there was a significant expression difference of *UBA1* between stages in testicular germ cell tumours (TGCT), READ and uveal melanoma (UVM), the expression level of *UBA1* gradually increased in UVM and DLBC (Figure S2A–D).

### 3.3 Pan‐cancer analysis of the prognostic value of UBA1

**FIGURE 3:**
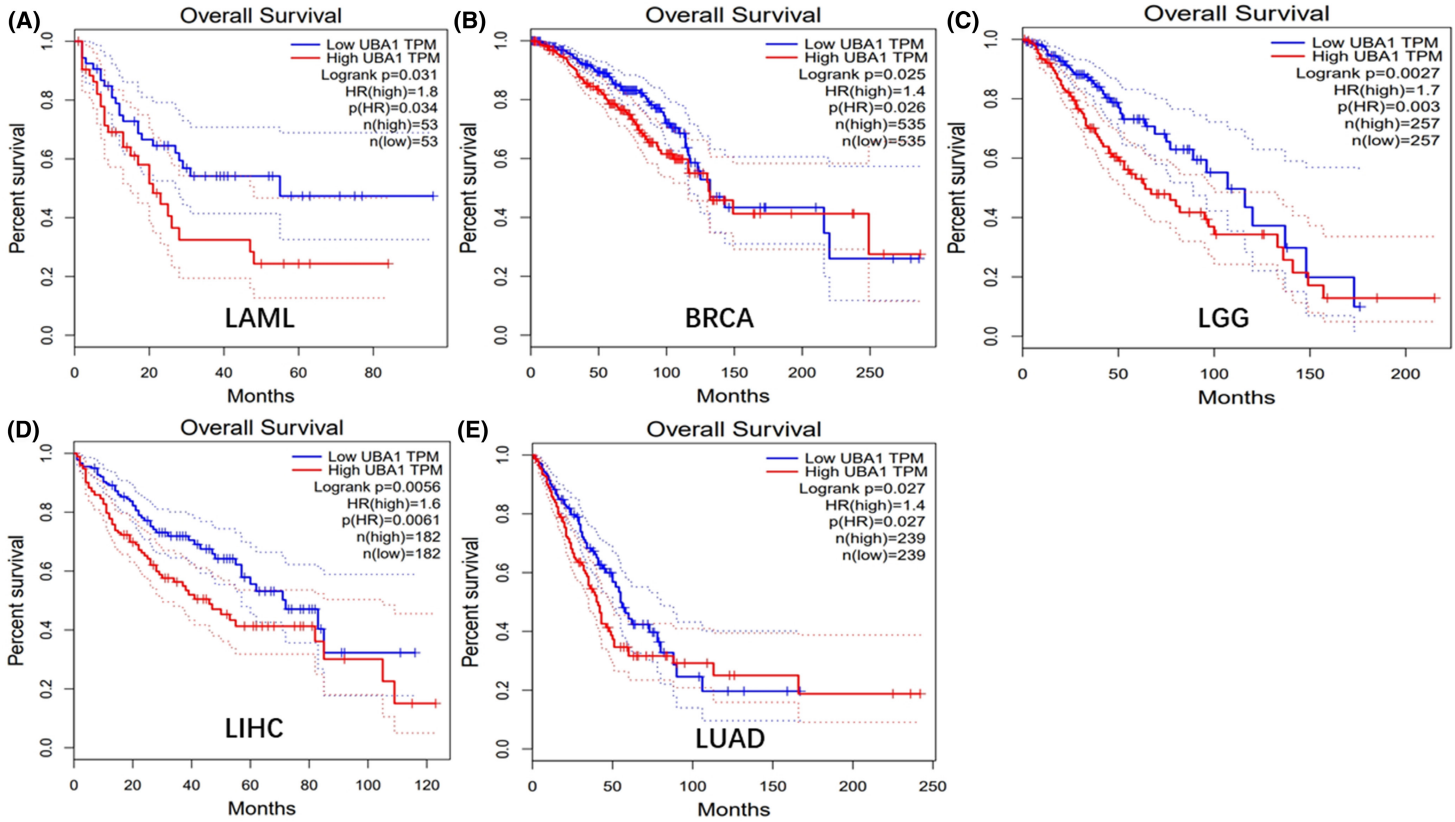
Pan‐cancer analysis of the prognostic value of *UBA1*.The Kaplan Meier survival curve showed that upregulation of *UBA1* expression was significantly associated with poor OS in LAML, BRCA, LGG, LIHC and LUAD (A–E).

The relationship between *UBA1* expression and the prognosis of cancer patients was estimated using clinical survival data from TCGA pan‐cancer. Survival characteristics include OS, PFS, DFS, and DSS. The Kaplan Meier survival curve showed that upregulation of *UBA1* expression was significantly associated with poor OS in LAML, BRCA, LGG, LIHC and LUAD (Figure 3A–E). Cox regression analysis of 33 types of cancer showed a significant correlation between *UBA1* expression and OS in 8 types of cancer, including bladder urothelial carcinoma (BLCA) (HR >1, *p* < 0.05), head and neck squamous cell carcinoma (HNSC) (HR >1, *p* < 0.05), KIRC (HR <1, *p* < 0.05), LAML (HR >1, *p* < 0.05), LGG (HR >1, *p* < 0.05), LIHC (HR >1, *p* < 0.05), lung adenocarcinoma (LUAD) (HR >1, *p* < 0.05) and THCA (HR <1, *p* < 0.05) (Figure S3A). *UBA1* expression is significantly correlated with PFS in four types of cancer, including adrenocortical carcinoma (ACC) (HR >1, *p* < 0.05), BLCA (HR >1, *p* < 0.05), LGG (HR >1, *p* < 0.05) and mesothelioma (MESO) (HR >1, *p* < 0.05) (Figure S3B). The expression of *UBA1* in 8 types of cancer is significantly correlated with DFS, including BLCA (HR >1, *p* < 0.05), HNSC (HR >1, *p* < 0.05), KIRC (HR <1, *p* < 0.05), LAML (HR >1, *p* < 0.05), LGG (HR >1, *p* < 0.05), LIHC (HR >1, *p* < 0.05), LUAD (HR >1, *p* < 0.05) and THCA (HR <1, *p* < 0.05) (Figure S3C). The expression of *UBA1* in three types of cancer is significantly correlated with DSS, including BLCA (HR >1, *p* < 0.05), LGG (HR >1, *p* < 0.05) and LUAD (HR >1, *p* < 0.05) (Figure S3D). We found that upregulation of *UBA1* expression was significantly correlated with poor OS, PFS, DFS and DSS in LGG patients.

### 3.4 UBA1 co‐expressed gene/protein interaction network and enrichment analysis

**FIGURE 4:**
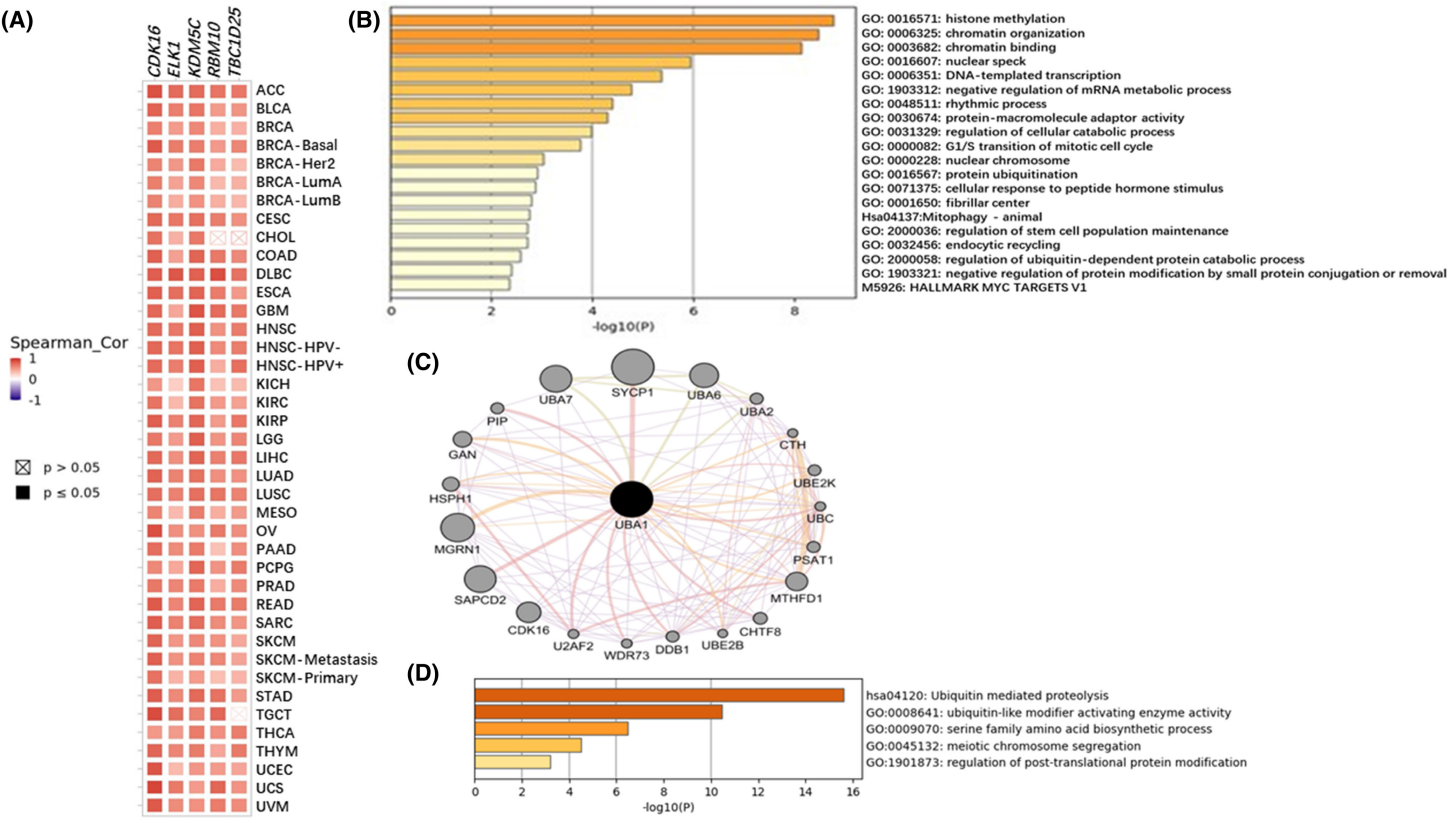
*UBA1* coexpressed gene/protein interaction network and enrichment analysis. Analysing the top 100 co‐expressed genes of *UBA1* in pan‐cancer on GEPIA2.0, the top 5 genes (*CDK16*, *ELK1*, *KDM5C*, *RBM10* and *TBC1D25*) were highly correlated with *UBA1* in most cancer types (A). The functional enrichment of GO exhibits activities related to histone methylation, negative regulation of mRNA metabolism, negative regulation of protein modification, DNA template transcription, protein ubiquitination, and cell cycle (B). Then, further research was conducted on the potential impact of *UBA1* on protein interaction networks, and 20 genes related to *UBA1* were extracted from the GeneMANIA database for GO and KEGG enrichment analysis (C). GO enrichment analysis showed that *UBA1* is involved in some pathways, including ubiquitin like modifier activating enzyme activity, serine family amino acid biosynthesis process, DNA damage response, and microtubule cytoskeletal tissue. In addition, KEGG pathway analysis showed that *UBA1* was involved in the ubiquitin mediated protein hydrolysis pathway (D).

Analysing the top 100 co‐expressed genes of *UBA1* in pan‐cancer on GEPIA2.0, the top 5 genes (*CDK16*, *ELK1*, *KDM5C*, *RBM10* and *TBC1D25*) were highly correlated with *UBA1* in most cancer types (Figure 4A). The functional enrichment of GO exhibits activities related to histone methylation, negative regulation of mRNA metabolism, negative regulation of protein modification, DNA template transcription, protein ubiquitination and cell cycle (Figure 4B). Then, further research was conducted on the potential impact of *UBA1* on protein interaction networks and 20 genes related to *UBA1* were extracted from the GeneMANIA database for GO and KEGG enrichment analysis (Figure 4C). GO enrichment analysis showed that *UBA1* is involved in some pathways, including ubiquitin like modifier activating enzyme activity, serine family amino acid biosynthesis process, DNA damage response, and microtubule cytoskeletal tissue. In addition, KEGG pathway analysis showed that *UBA1* was involved in the ubiquitin mediated protein hydrolysis pathway (Figure 4D).

### 3.5 Pan‐cancer analysis of methylation levels and UBA1 mutations

According to the UALCAN database, the levels of *UBA1* methylation in prostate adenocarcinoma (PRAD), TGCT, BLCA, LIHC, KIRC and THCA tissues significantly decreased compared to normal tissues (Figure S4A–F). We used the cBioPortal database to study the mutation of *UBA1* in pan‐cancer. The results show that the highest mutation frequency of *UBA1* in bladder cancer patients is about 27% (Figure S5A). Among different types of gene mutations, deletion is the most common. We also investigated the potential relationship between gene mutations in *UBA1* and the prognosis of patients with different types of cancer (Figure S6A). As shown in Figure S6A, compared with patients without mutations, tumour patients with *UBA1* gene mutations have significantly poorer prognosis in OS.

### 3.6 Pan‐cancer analysis of UBA1 phosphorylation

Based on the CPTAC database, we conducted phosphorylation analysis and the analysis results showed that compared with normal samples, LUAD, lung squamous cell carcinoma (LUSC), PAAD and LIHC had higher phosphorylation degree in S46 site of *UBA1* protein (Figure S7A–D) and kidney renal clear cell carcinoma (Clear cell RCC) had lower phosphorylation degree in S46 site of *UBA1* protein (Figure S7E).

### 3.7 Pan‐cancer analysis of the correlation between UBA1 expression and immune regulatory factors TMB, MSI and MMR related genes

We investigated the association between *UBA1* expression and TMB and MSI, both of which involve sensitivity to immune checkpoint blockade. Therefore, it is necessary to study the relationship between TMB and *UBA1* in cancer. *UBA1* expression was significantly correlated with TMB in 10 types of cancer (*p* < 0.05). *UBA1* was positively correlated with TMB in 9 types of tumours including STAD, UCEC, sarcoma (SARC), skin cutaneous melanoma (SKCM), PAAD, GBM, LUSC and LGG, while negatively correlated with TMB in THCA and KIRP (Figure S8A). *UBA1* expression was significantly correlated with MSI in 11 types of cancer (*p* < 0.05). *UBA1* is positively correlated with MSI in 8 types of tumours, including LUSC, KIRC, STAD, UCEC, UVM, TGCT, LUAD and LIHC, while negatively correlated with MSI in DLBC, pheochromocytoma and paraganglioma (PCPG) and READ (Figure S8B). Then, the correlation between *UBA1* expression and MMR genes was evaluated, including *MLH1*, *MSH2*, *MSH6*, *PMS2* and *EPCAM* (Figure S8C). Figure S8C illustrates the correlation between *UBA1* expression and the expression of a single MMR gene. Except for READ, SKCM, uterine carcinosarcoma (UCS) and UVM, the expression of *UBA1* in most tumours is associated with the expression of the MMR gene.

### 3.8 UBA1 and immune infiltration, single‐cell, immune checkpoint and immune factors

**FIGURE 5:**
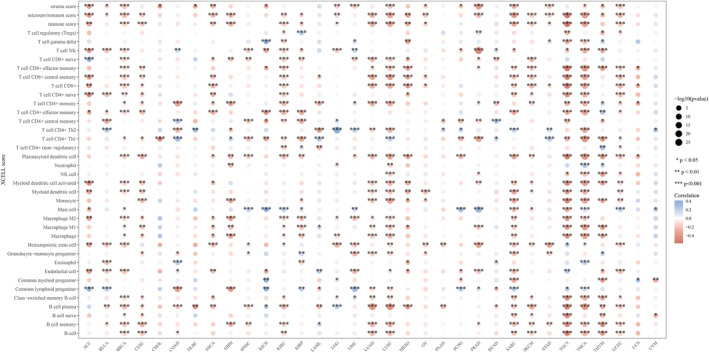
*UBA1* and immune infiltration. *UBA1* is positively and significantly correlated with matrix score, ImmuneScore and microenvironment score in many cancers. In most tumours, *UBA1* expression also shows a significant positive correlation with immune cell infiltration. *UBA1* showed a significant positive correlation with immune score, immune microenvironment score, Neutrophil, MacrophageM2 and Macrophage in GBM and LGG, a significant positive correlation with MacrophageM1 in GBM and a positive correlation with MacrophageM1 in LGG. There is a significant positive correlation with T cell CD4 + Th2 in GBM and a significant negative correlation with T cell CD4 + Th2 in LGG. UBA1 is significantly positively correlated with immune cell infiltration in CESC, CHOL, ESCA, LUAD, MESO, OV and SKCM.

**FIGURE 6:**
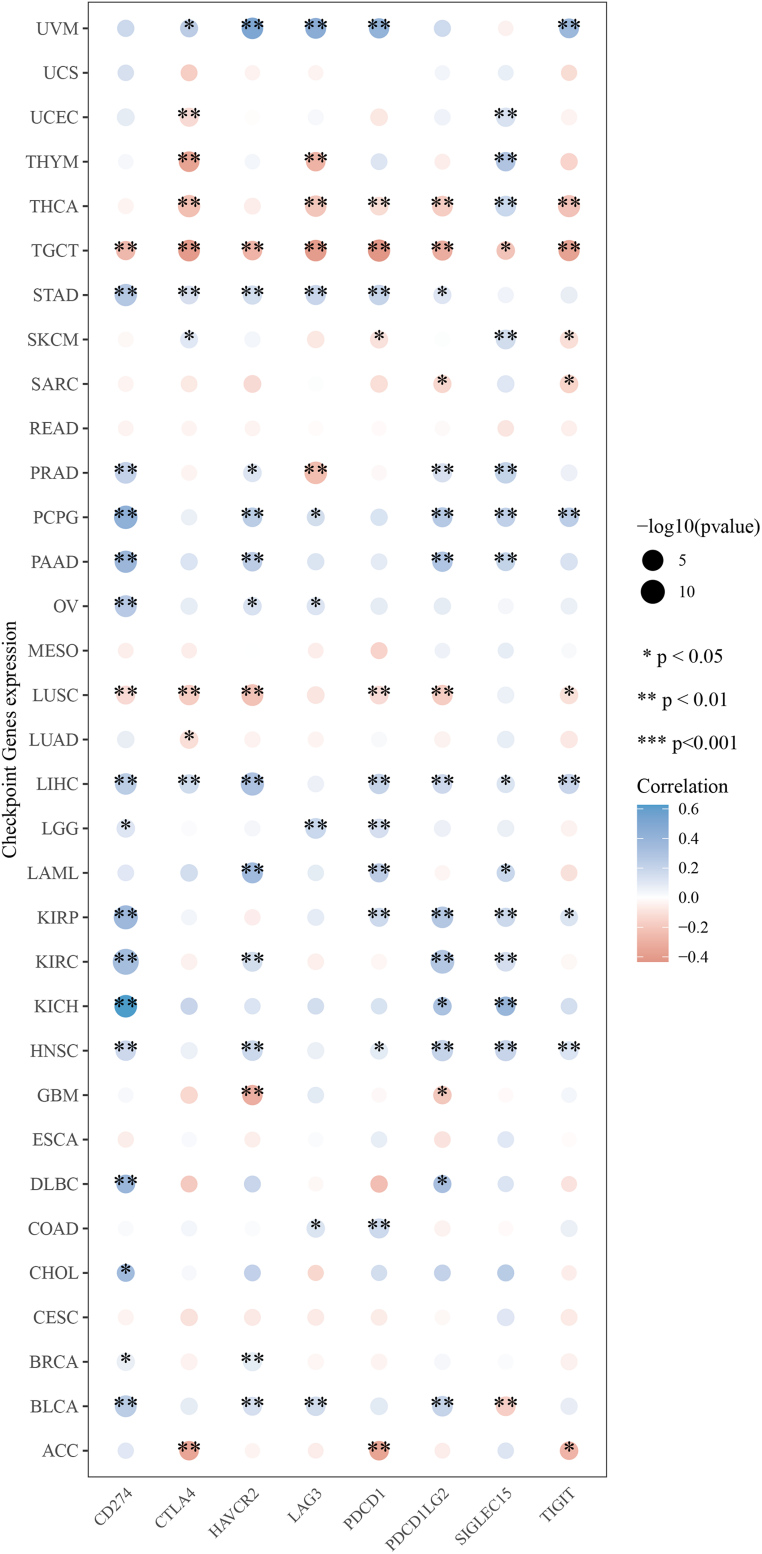
*UBA1* and immune checkpoint. In TGCT, *UBA1* was significantly positively correlated with 8 immune checkpoint genes.

In order to investigate the immunological role of *UBA1* in cancer environments, the estimated values of *UBA1* in pan‐cancer were calculated (Figure 5). As shown in Figure 5, *UBA1* is positively and significantly correlated with matrix score, ImmuneScore and microenvironment score in many cancers. In most tumours, *UBA1* expression also shows a significant positive correlation with immune cell infiltration. *UBA1* showed a significant positive correlation with immune score, immune microenvironment score, Neutrophil, MacrophageM2 and Macrophage in GBM and LGG, a significant positive correlation with MacrophageM1 in GBM and a positive correlation with MacrophageM1 in LGG. There is a significant positive correlation with T cell CD4 + Th2 in GBM and a significant negative correlation with T cell CD4 + Th2 in LGG. UBA1 is significantly positively correlated with immune cell infiltration in cervical squamous cell carcinoma (CESC), cholangiocarcinoma (CHOL), ESCA, LUAD, MESO, OV and SKCM. Retrieved *UBA1* expression data from TISCH for single‐cell subtypes (Figure S9). As shown in Figure S9, *UBA1* is expressed by macrophages and malignant cells in BRCA, DLBC, glioma, LIHC, OV and PAAD. In TGCT, *UBA1* was significantly positively correlated with 8 immune checkpoint genes (Figure 6). Subsequently, we investigated whether *UBA1* is differentially expressed in different cancer immune subtypes through the TISIDB database. The histogram shows a significant correlation (*p* < 0.01) between *UBA1* and the immune subtypes of 10 types of cancer (Figure S10A) and we present the top three cancer types (Figure S10B). We analysed the association between *UBA1* and chemokines, receptors and immunostimulants. As shown in the heatmap, *UBA1* is negatively correlated with chemokines, many receptors and immune stimulatory factors in pan‐cancer (Figure S10C).

### 3.9 UBA1 predicts treatment response to Pan‐cancer

In order to investigate whether *UBA1* can predict treatment response to cancer, data was obtained from ROCplotter to demonstrate the association between treatment outcomes and *UBA1* expression for four types of cancer (BRCA, OV, GBM, and CRC). Respondents treated with nivolumab immunotherapy showed higher expression of *UBA1*, with an area under curve (AUC) value of 0.65 (Figure S11A). Respondents treated with PD1 immunotherapy showed higher expression of *UBA1*, with an AUC value of 0.58 (Figure S11B). In CRC, responders treated with fluoropyrimidine monotherapy showed higher *UBA1* expression, with an AUC value of 0.69 (Figure S11C). In OV, responders treated with docetaxel exhibited higher *UBA1* expression, with an AUC value of 0.77 (Figure S11D). In GBM, *UBA1* is highly expressed in post chemotherapy responders, with an AUC value of 0.60 (Figure S11E). However, in BRCA, responders after endocrine therapy had higher *UBA1* expression, with an AUC value of 0.58 (Figure S11F). The drug sensitivity of *UBA1* expression in tumours was studied using GSCALite. The expression of *UBA1* is positively correlated with the 50% inhibitory concentration (IC50) values of CAMPTOTHECIN, sr.13654 and Arsenal (Figure S11G).

### 3.10 Construction of UBA1 overexpression DLBCL cell model and UBA1 interference AML cell model

The qPCR results showed that compared with the OCI‐LY1 + NC, the cells transfected with OCI‐LY1 + *UBA1*‐Overexpression had the significant overexpression effect on *UBA1* and named OCI‐LY1 + *UBA1*‐Overexpression (Figure S12A). The qPCR results showed that compared with the *UBA1*‐NC, the cells transfected with HL‐60 + *UBA1*‐siRNA1 had the most significant interference effect on *UBA1* (Figure S12B). The cells transfected with HL‐60 + *UBA1*‐siRNA1 were therefore selected as the cell model for *UBA1* interference and named HL‐60 + *UBA1*‐siRNA1(1036).

### 3.11 Cycle experiments of DLBCL cells

**FIGURE 7:**
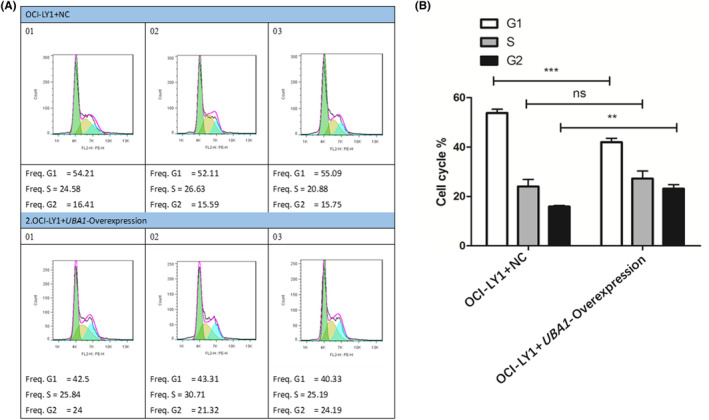
Cycle experiments of DLBCL cells. The results of cell proliferation showed that compared with the OCI‐LY1 + NC group cells, the OCI‐LY1 + *UBA1*‐Overexpression group showed a significant increase in G1 phase cells decreased, S phase cells showed no significant changes and G2 phase cells increased (A, B). Therefore, the high expression of UBA1 significantly promotes the progression of DLBCL cell cycle.

The results of cell proliferation showed that compared with the OCI‐LY1 + NC group cells, the OCI‐LY1 + *UBA1*‐Overexpression group showed a significant increase in G1 phase cells decreased, S phase cells showed no significant changes, and G2 phase cells increased (Figure 7A,B). Therefore, the high expression of UBA1 significantly promotes the progression of DLBCL cell cycle.

### 3.12 Apoptosis experiments of DLBCL cells

**FIGURE 8:**
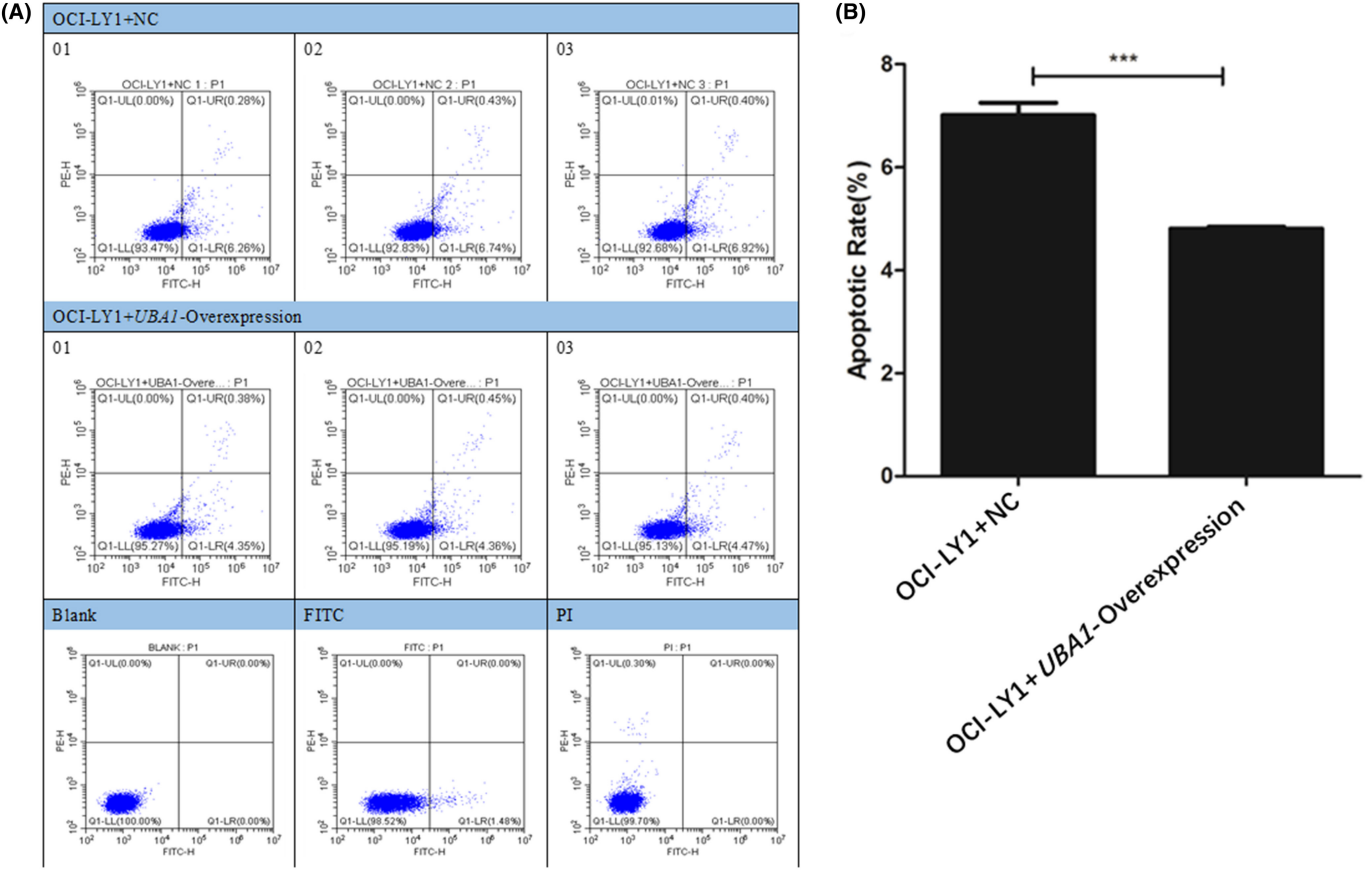
Apoptosis experiments of DLBCL cells. The results of cell apoptosis showed that compared with the OCI‐LY1 + NC group cells, the apoptosis rate of the OCI‐LY1 + *UBA1*‐Overexpression group cells were significantly reduced (A, B). Therefore, the high expression of *UBA1* significantly inhibited apoptosis of DLBCL cells.

The results of cell apoptosis showed that compared with the OCI‐LY1 + NC group cells, the apoptosis rate of the OCI‐LY1 + *UBA1*‐Overexpression group cells were significantly reduced (Figure 8A,B). Therefore, the high expression of *UBA1* significantly inhibited apoptosis of DLBCL cells.

### 3.13 Cycle experiments of AML cells

**FIGURE 9:**
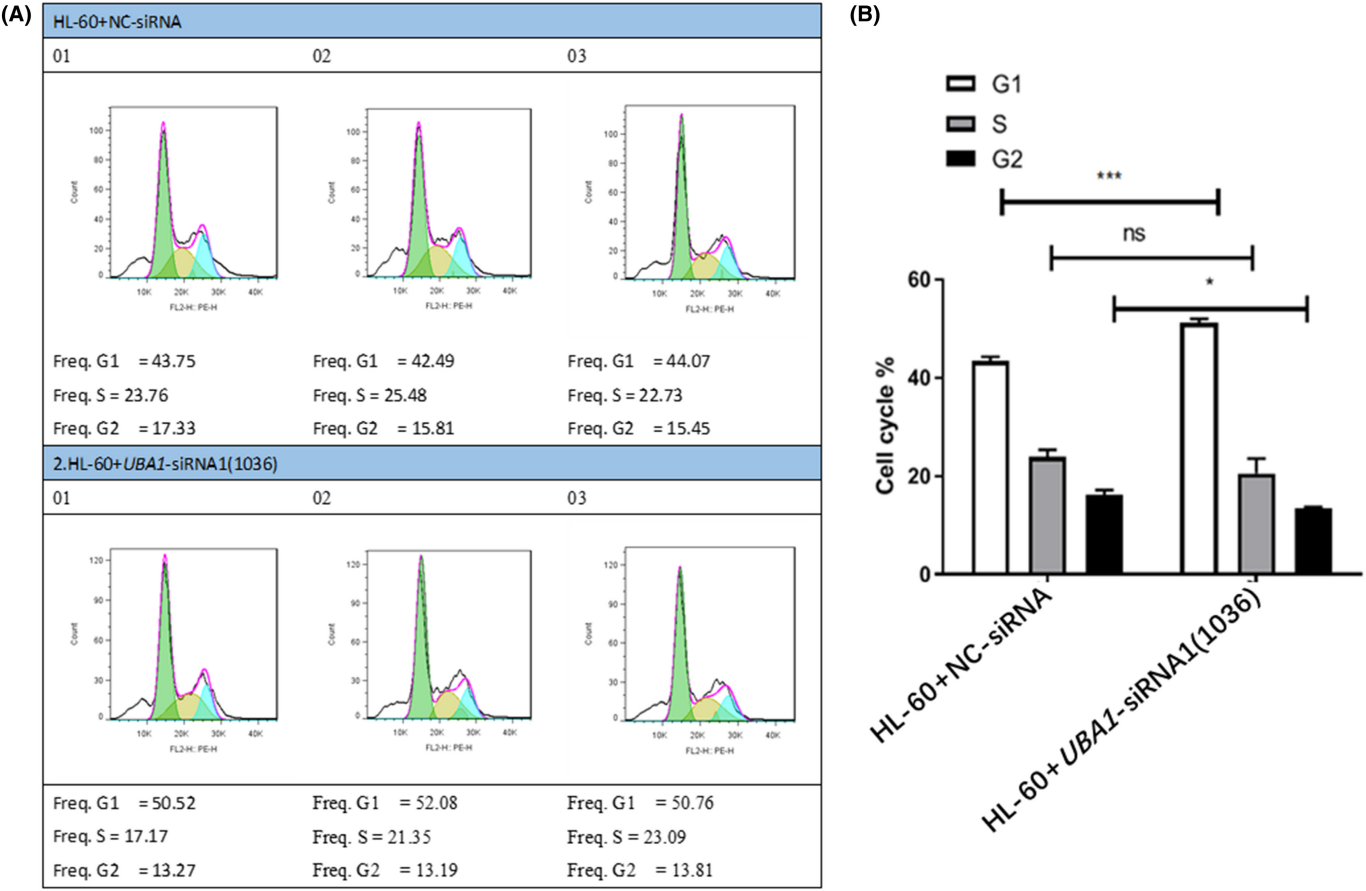
Cycle experiments of AML cells. The results of cell proliferation showed that compared with the HL‐60 + NC‐siRNA group cells, the HL‐60 + *UBA1*‐siRNA1 group showed a significant increase in G1 phase cells increase, S phase cells remain unchanged and G2 phase cells decrease (A, B). Therefore, the low expression of *UBA1* significantly inhibited the progression of AML cell cycle.

The results of cell proliferation showed that compared with the HL‐60 + NC‐siRNA group cells, the HL‐60 + *UBA1*‐siRNA1 group showed a significant increase in G1 phase cells increase, S phase cells remain unchanged and G2 phase cells decrease (Figure 9A,B). Therefore, the low expression of *UBA1* significantly inhibited the progression of AML cell cycle.

### 3.14 Apoptosis experiments of AML cells

**FIGURE 10:**
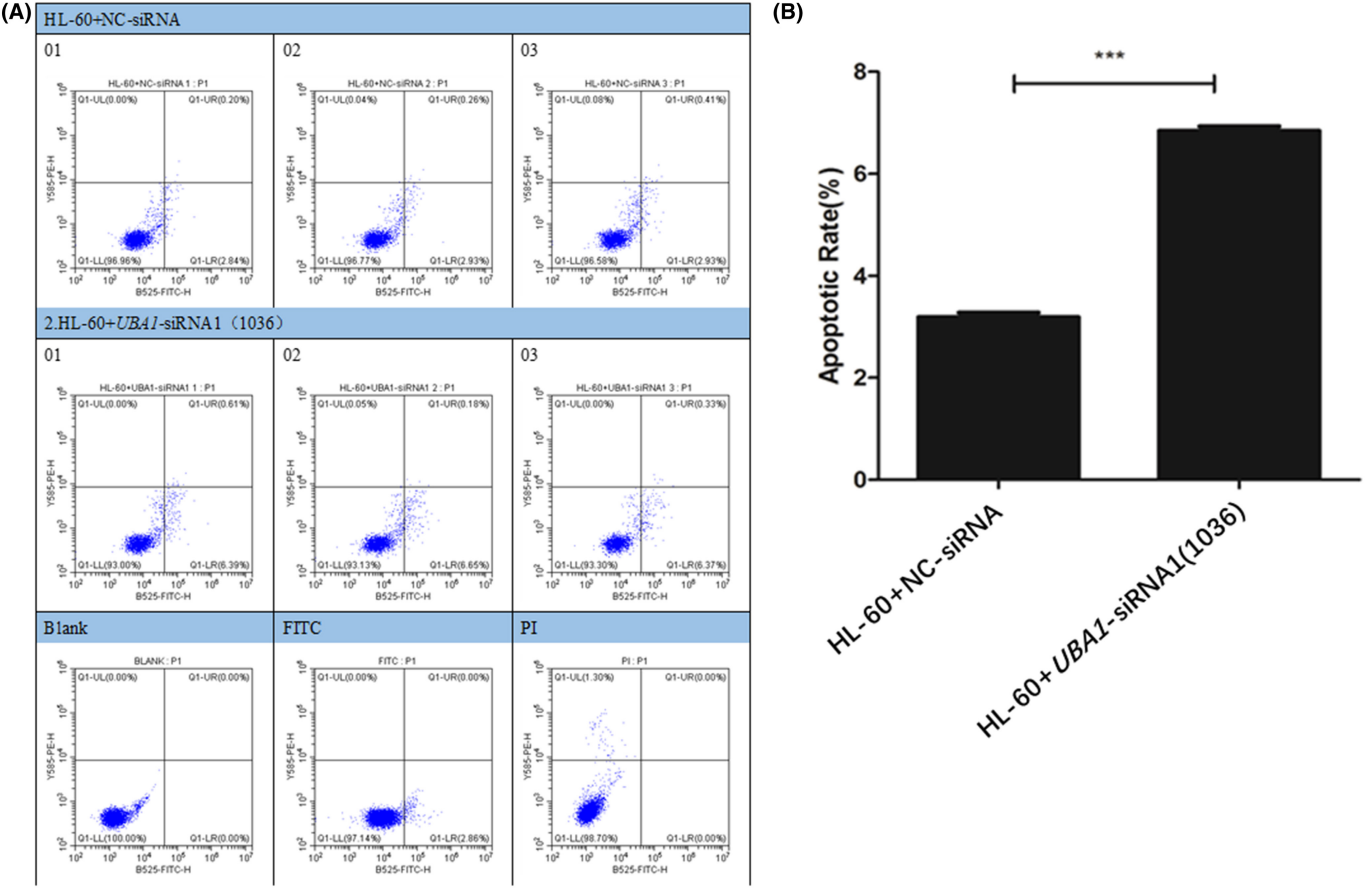
Apoptosis experiments of AML cells. The results of cell apoptosis showed that compared with the HL‐60 + NC‐siRNA group cells, the apoptosis rate of the HL‐60 + *UBA1*‐siRNA1 group cells was significantly increased (A, B). Therefore, the low expression of *UBA1* significantly promotes apoptosis of AML cells.

The results of cell apoptosis showed that compared with the HL‐60 + NC‐siRNA group cells, the apoptosis rate of the HL‐60 + *UBA1*‐siRNA1 group cells was significantly increased (Figure 10A,B). Therefore, the low expression of *UBA1* significantly promotes apoptosis of AML cells.

## 4 DISCUSSION

Ubiquitin is a small molecule protein composed of 76 amino acids with a molecular weight of approximately 8.5 kDa, widely present in all eukaryotic cells.^42^ Ubiquitination refers to the multi‐step process in which ubiquitin covalently binds to target proteins under the catalytic action of a series of ubiquitinases, which is crucial for regulating a large number of cellular processes.^43^ Ubiquitination is strictly regulated by a series of enzymes at different levels, including ubiquitin activating enzyme E1, ubiquitin binding enzyme E2 and ubiquitin ligase E3.^44^ Ubiquitination plays a crucial role in tumour necrosis factor (TNF) mediated inflammatory signalling and cell death regulation.^45^ TNF is the most extensively studied member of the inflammatory cytokine TNF family, mediating the expression of NF—κ B and MAPK pathway related genes, inducing apoptosis and necrotic cell death.^46^ *UBA1* is the main ubiquitin activating enzyme E1, which can synergistically interact with 30 different E2s and approximately 600 different E3s, catalysing the ubiquitination of hundreds of substrate proteins, thereby regulating a wide range of cellular functions.^47^ In recent years, it has been proven to be associated with cancer.^48^ However, the pathological mechanism of *UBA1* in pan‐cancer is not fully understood. To our knowledge, this study provides the first pan‐cancer analysis of *UBA1* in tumours, which will provide new ideas and directions for further research on the functional role of ubiquitination in tumours.

The changes in expression levels in tumour tissues are a prerequisite for genes to play important regulatory functions. Through analysing TCGA and GTEx data, we found that *UBA1* is significantly overexpressed in gastrointestinal tumours (COAD, ESCA, LIHC, PAAD, READ and STAD), haematological tumours (DLBCL and LAML/AML) and brain tumours (LGG and GBM). Subsequent OS, PFS, DFS and DSS analyses also showed that the expression of *UBA1* is closely related to the clinical prognosis of various cancers, especially LAML and LGG. The DNA methylation data and protein phosphorylation data from the UALCAN database further support the important role of *UBA1* in pan‐cancer. Protein phosphorylation plays a crucial role in studying the molecular mechanisms of tumorigenesis and cancer progression.^49^ The high degree of phosphorylation of protein sites suggests that phosphorylation of this protein site may play a promoting role in the occurrence and development of cancer.^50^ Our analysis results show that LUAD, LUSC, PAAD and LIHC had higher phosphorylation degree in S46 site of *UBA1* protein, revealing that protein phosphorylation of *UBA1* at S46 site might serve as a facilitator in the development and progression of these cancers. Our analysis results show that high expression of *UBA1* is significantly correlated with poor OS prognosis in LIHC and LUAD (HR >1 and *p* < 0.05). Therefore, the high degree of phosphorylation at the S46 site of *UBA1* protein may be one of the reasons for poor prognosis in pan cancer. The clinical pathological staging analysis showed that the expression level of *UBA1* increased with the progression of DLBCL disease. Therefore, *UBA1* may play an important role in haematological tumours (DLBCL and AML), which are one of the leading causes of cancer death worldwide.^51^ Our research confirms that high expression of *UBA1* significantly promotes the progression of DLBCL cell cycle, while low expression of *UBA1* significantly inhibits the progression of AML cell cycle. These results fully support our speculation that *UBA1* is an important biomarker for haematological tumours. Higher expression of *UBA1* significantly promotes cell cycle progression and significantly inhibits apoptosis in haematological malignancies (DLBCL and AML). Prognostic results show that high expression of *UBA1* is a prognostic risk factor for OS in haematological malignancies (DLBCL and AML) (HR >1). Therefore, higher expression of *UBA1* may affect the prognosis of haematological malignancies (DLBCL and AML) by promoting sustained cell proliferation, inhibiting cell apoptosis and ultimately leading to tumour formation.

VEXAS syndrome is an inflammatory disease caused by *UBA1* gene somatic mutations, which is an adult‐onset inflammatory disease with overlapping haematological manifestations. Inflammatory response is one of the results of a strong immune response.^52^ A study has found that innate immune cells play an important role in the inflammatory process of VEXAS syndrome.^53^ At present, research on the immune regulation of *UBA1* mainly focuses on VEXAS syndrome.^54^ Our pan‐cancer study comprehensively analysed the potential role of *UBA1* in pan‐cancer immune regulation and predictive immunotherapy. MMR is a form of DNA repair mechanism in the body, mainly correcting base mismatches, preventing gene mutations and maintaining genomic stability. MMR functional defects are usually caused by pathogenic mutations in MMR genes (*MLH1, MSH2, MSH6*, and *PMS2*) and related gene *EPCAM*. *EPCAM* is widely expressed in various malignant tumours, especially in digestive system tumour cells. *EPCAM* is not an MMR gene, but it is an upstream gene of *MSH2*. Lack of *EPCAM* can lead to epigenetic inactivation of *MSH2*, resulting in *MSH2* silencing and disruption of the MMR pathway.^31^ MSI is a genetic mutation state caused by defects in DNA MMR function, characterized by the accumulation of microsatellite mutations. MSI is phenotypic evidence of abnormal MMR function.^55^ TMB refers to the number of somatic mutations per million DNA bases in the tumour genome after removing germline mutations. The higher the tumour TMB, the more new antigens the tumour produces, and the stronger the T cell response and anti‐tumour response.^56^ Our study found that the expression of *UBAl* is significantly correlated with MSI, TMB and MMR related genes (*MLH1, MSH2, MSH6, PMS2*, and *EPCAM*) in various cancers. In addition, in many cancers, *UBA1* is positively correlated with matrix score, immune score and microenvironment score. In most tumours, the expression of *UBAl* is also significantly positively correlated with immune cell infiltration. At the single‐cell level, *UBA1* is highly expressed in malignant cells and macrophages in various cancers (BRCA, DLBC, glioma, LIHC, OV and PAAD). *UBA1* is closely related to immune checkpoint genes in most cancers, and in TGCT, *UBA1* is significantly positively correlated with 8 immune checkpoint genes. Chemokines are a group of relatively small‐molecular‐weight secreted proteins that induce immune cell function and movement by interacting with chemokine receptors.^57^ Our analysis indicates that *UBA1* is closely related to the expression of chemokine receptors and chemokines in various cancers. These results indicate that *UBA1* is likely necessary for various tumour immunotherapies. There is a complex interrelationship between ubiquitination and cytokines.^58^ On the one hand, ubiquitination can regulate the stability and activity of cytokine signalling pathways, on the other hand, ubiquitination modification can promote the internalization and degradation of cytokine receptors, thereby regulating the duration and intensity of signal transduction.^59^ In addition, some ubiquitin ligases can also directly interact with cytokines to regulate their stability and function.^60^ Therefore, the abnormal expression of *UBA1* may also regulate the stability and function of certain cytokines to regulate the immune escape process of tumour cells, which will be the molecular mechanism to be explored in our further research.

In summary, this study reveals the important role of *UBA1* in pan‐cancer and validates its regulatory role in haematological tumours, providing new ideas and theoretical basis for the value of *UBA1* in tumour treatment.

This study inevitably has several limitations. This study mainly comes from public databases and is retrospective, there are potential confounding factors that require prospective research for further exploration. The expression level of *UBA1* may be influenced by clinical parameters and the number of clinical information datasets available for cancer patients in this study is limited, therefore, the expression level of *UBA1* in some cancer patients may not be accurate. Our study confirms that the expression of *UBA1* at the transcriptional level can regulate the function of DLBCL and AML cells. However, our experiment is limited to the impact of *UBA1* expression at the transcriptional level on cell function and its downstream and upstream mechanisms are not yet clear. The function of genes is influenced by various factors, for example, certain upstream miRNAs can regulate the transcriptional expression level of *UBA1*, thereby affecting its function, the functional mechanism of *UBA1* at the protein level has not been elucidated, therefore, further research is needed to fully elucidate the role of *UBA1*. The role of *UBA1* may vary among different types of cancer. Although research has identified the association between *UBA1* expression and clinical parameters of specific cancer types, extending these findings to all cancer types may oversimplify the complex biological landscape of cancer. In addition, in vitro cell experiments do not involve interactions between cells in vivo or regulation of neuroendocrine systems. In vitro experiments cannot fully represent the characteristics of the tissue from which the cells originate. Therefore, further animal experiments are needed for further research.

## 5 CONCLUSIONS

In summary, our study conducted a comprehensive investigation into the *UBA1* mRNA expression characteristics, its prognostic value, and its relationship with tumour‐infiltrating immune cells across various cancers, utilizing a multi‐omic bioinformatics approach. We also validated the functional mechanisms of *UBA1* expression in haematological tumours, specifically DLBCL and AML. These findings underscore the crucial role of *UBA1* expression in the prognosis and treatment of cancer, highlighting significant opportunities for further research and validation to enhance therapeutic strategies and patient outcomes.

## AUTHOR CONTRIBUTIONS

**Can Chen:** Formal analysis (lead); investigation (lead); validation (lead). **Yiwei Li:** Formal analysis (supporting); investigation (supporting). **Zhenzhen Chen:** Formal analysis (supporting). **Pengfei Shi:** Writing – original draft (supporting). **Yun Li:** Investigation (supporting); writing – original draft (lead). **Shenxian Qian:** Funding acquisition (supporting); software (lead); writing – original draft (equal).

## FUNDING INFORMATION

This study was funded by Hangzhou science and technology Major Project with Grant Number: 202004A15, Hangzhou Medical health science and technology Major Project with Grant Number: Z20210039 and Zhejiang Province Traditional Chinese medicine science and technology project with Grant Number: 2023ZR122. Zhejiang Provincial Medical and Health Science and Technology Plan Project with Grant Number: 2021ky881.

## CONFLICT OF INTEREST

No conflicts of interest declared.

## CONSENT

Not applicable.

## Supporting information


Appendix S1.

## Data Availability

Gene expression datasets are publicly available (TCGA database (https://www.cancer.gov/ccg/research/genome‐sequencing/tcga) and GEO dataset (https://www.ncbi.nlm.nih.gov/geo/)).
