# Supplementary material for: A comprehensive prognostic and immune infiltration analysis of UBA1 in pan‐cancer: A computational analysis and in vitro experiments
Source: J Cell Mol Med. 2024 Aug 25;28(16):e70037. doi: 10.1111/jcmm.70037 (PMC11345122; doi:10.1111/jcmm.70037)
Supplement: Supplementary file 1 — Appendix S1. [file JCMM-28-e70037-s001.docx]

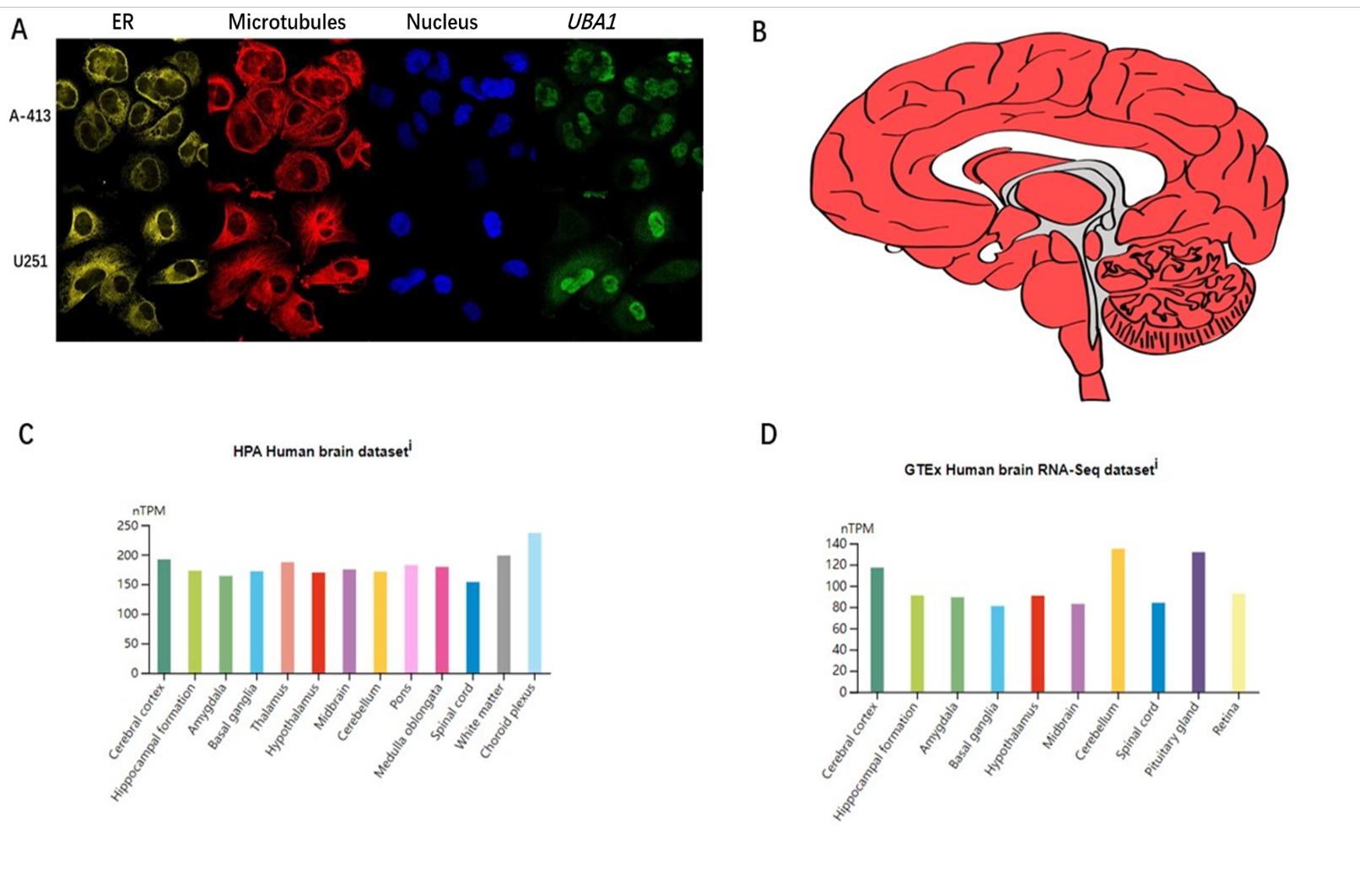


**Figure S1 Subcellular localization of *UBA1* and its expression distribution in the brain**

(A) Based on the HPA database, the subcellular localization of *UBA1* was obtained through immunofluorescence localization of nuclei, microtubules and ER in A-431 and U-251 MG cells. *UBA1* is mainly located in Nucleopasm;

(B-D) Demonstrated the expression level and distribution of *UBA1* in the brain.


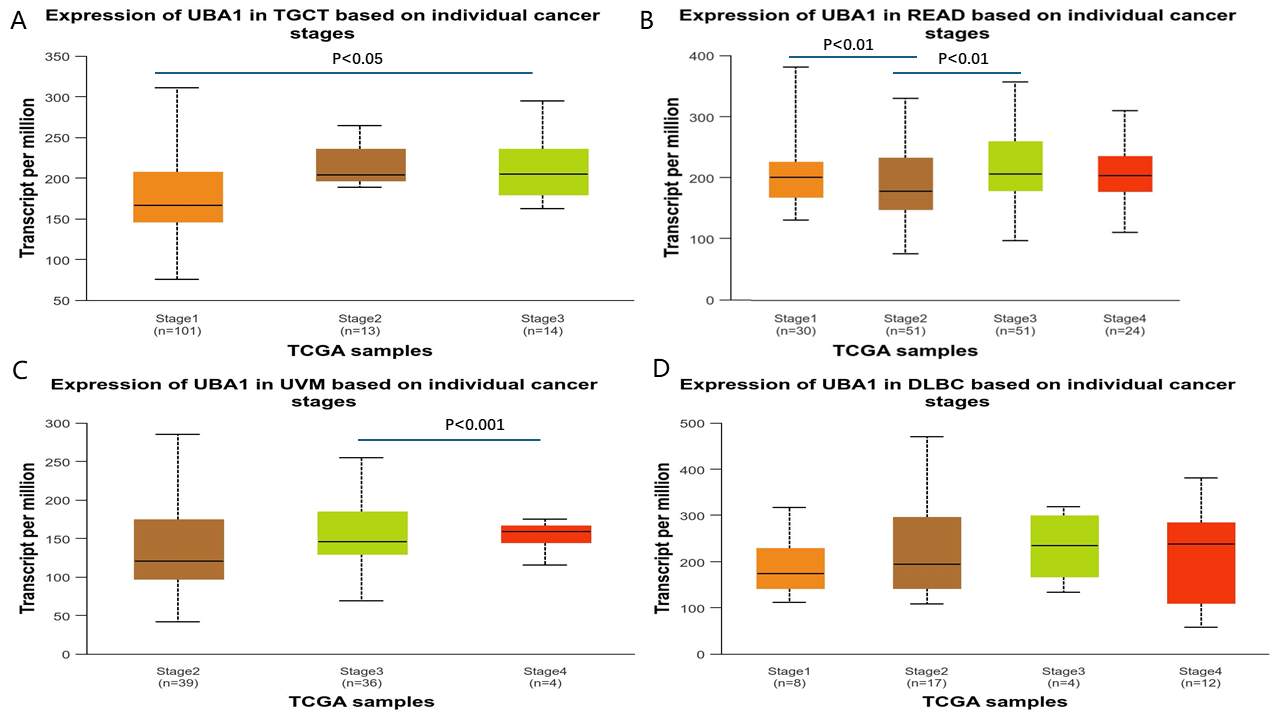


**Figure S2 Pan-cancer analysis of the correlation between** ***UBA1* expression and clinical stages**

(A) The results from the TCGA database showed that there was a significant expression difference of *UBA1* between stages in TGCT.

(B) The results from the TCGA database showed that there was a significant expression difference of *UBA1* between stages in READ.

(C) The results from the TCGA database showed that there was a significant expression difference of *UBA1* between stages in UVM.

(D) The expression level of *UBA1* gradually increased in DLBC.


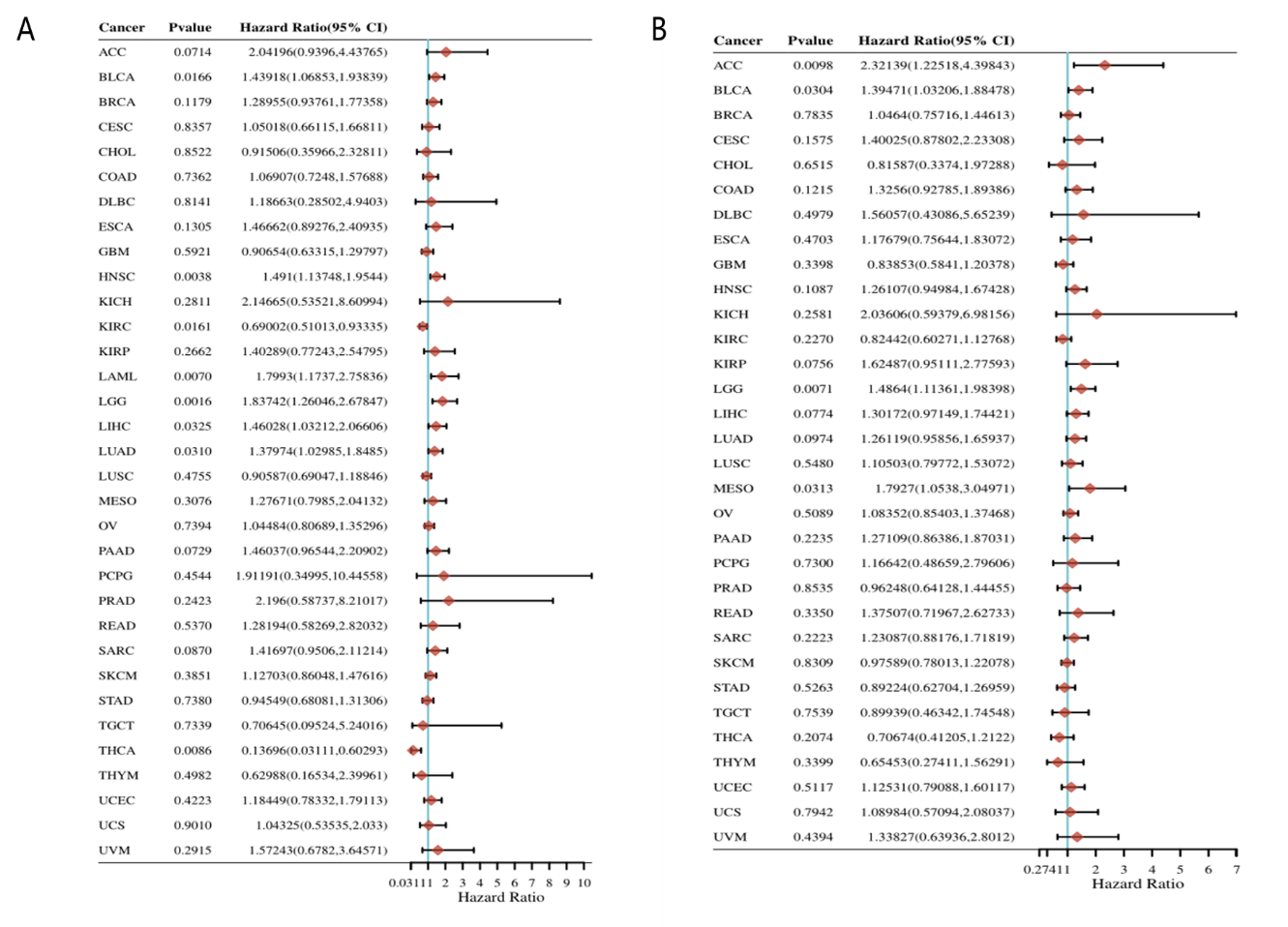


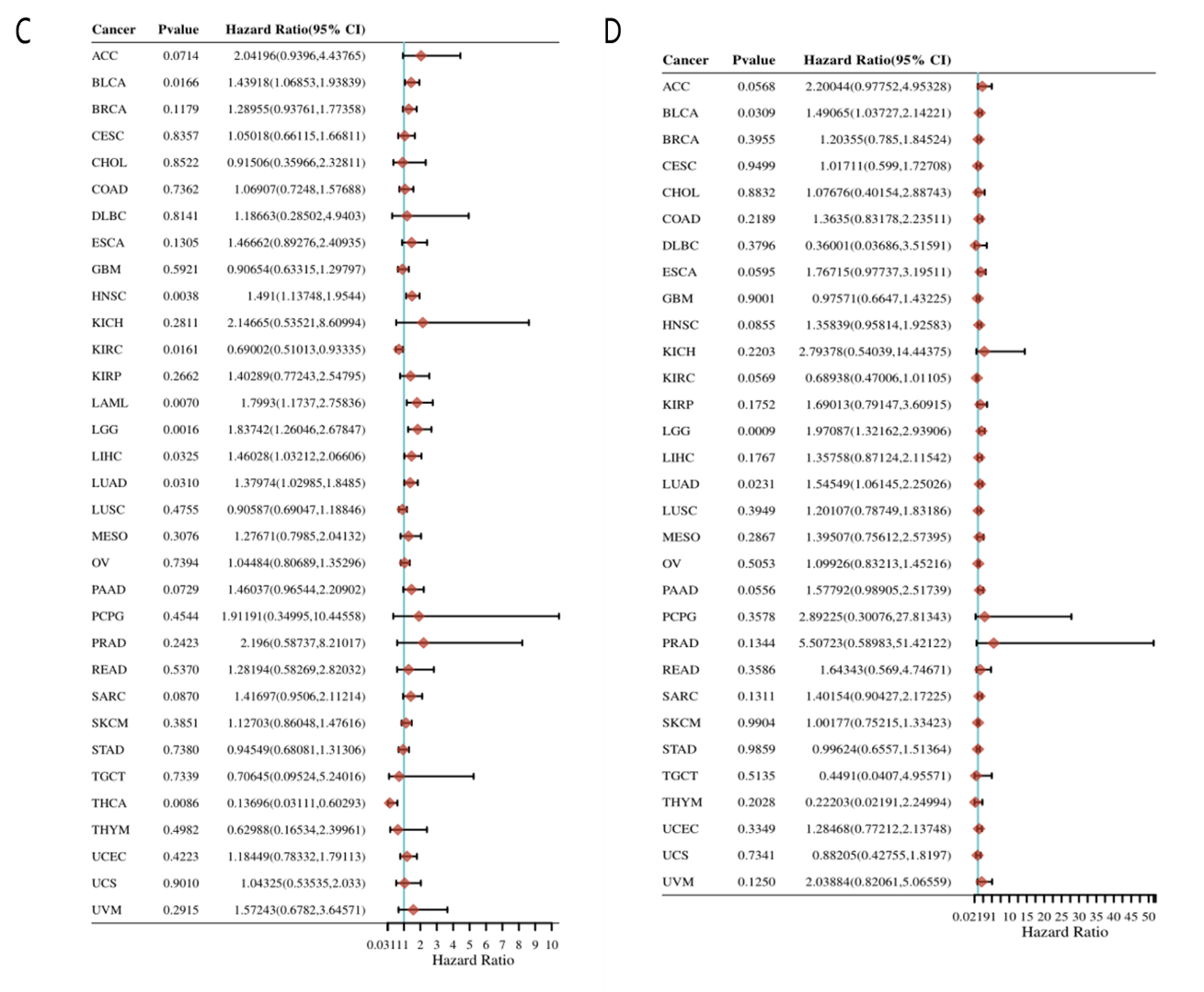


**Figure S3 Cox regression analysis was conducted on the prognostic characteristics of 33 types of cancer based on the TCGA database**

(A) Cox regression analysis–OS;

(B) Cox regression analysis–PFS;

(C) Cox regression analysis–DFS;

(D) Cox regression analysis–DSS.


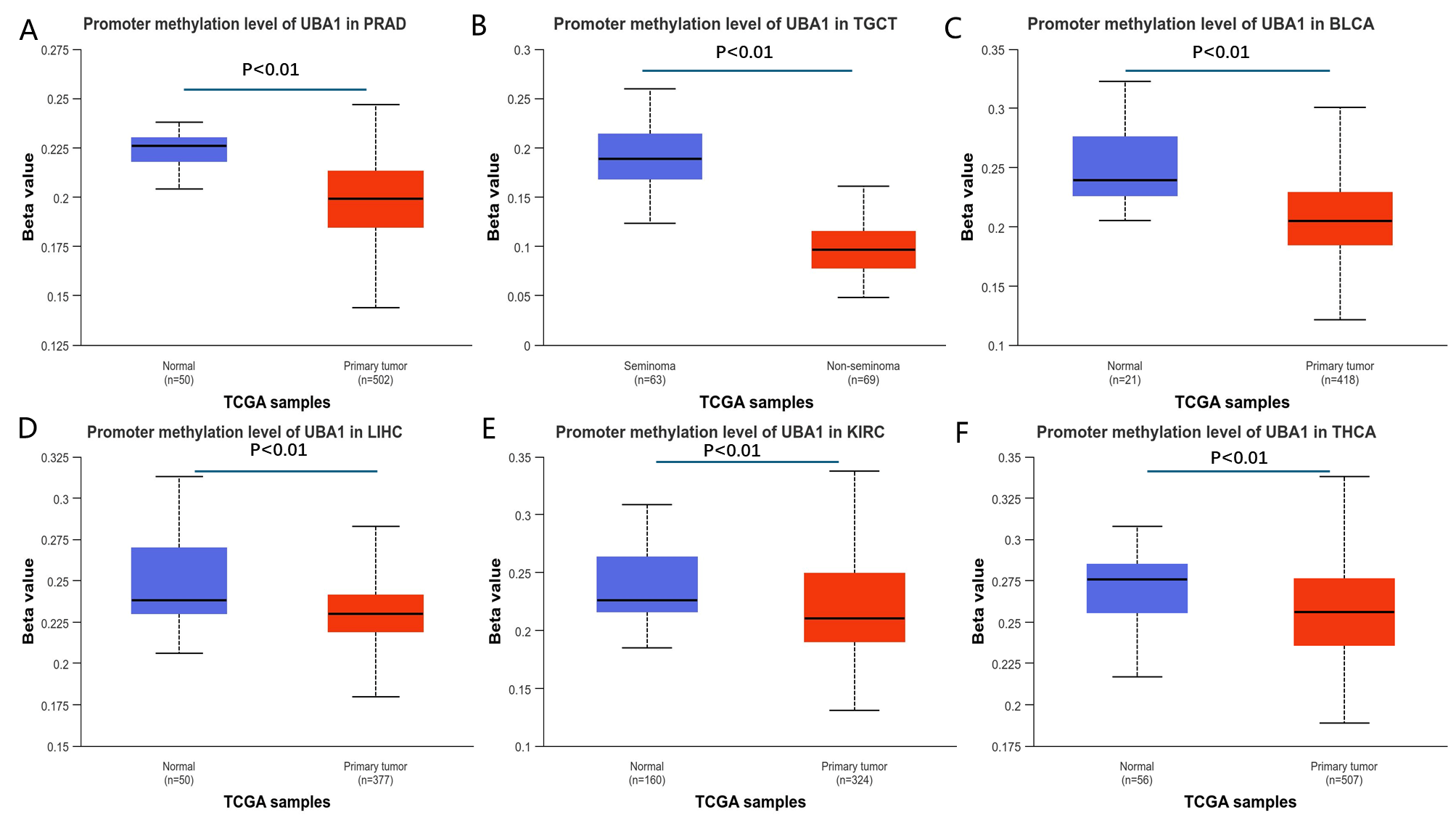


**Figure S4 Pan-cancer analysis of methylation levels and *UBA1* mutations**

(A) According to the UALCAN database, the levels of *UBA1* methylation in PRAD tissues significantly decreased compared to normal tissues.

(B) According to the UALCAN database, the levels of *UBA1* methylation in TGCT tissues significantly decreased compared to normal tissues.

(C) According to the UALCAN database, the levels of *UBA1* methylation in BLCA tissues significantly decreased compared to normal tissues.

(D) According to the UALCAN database, the levels of *UBA1* methylation in LIHC tissues significantly decreased compared to normal tissues.

(E) According to the UALCAN database, the levels of *UBA1* methylation in KIRC tissues significantly decreased compared to normal tissues.

(F) According to the UALCAN database, the levels of *UBA1* methylation in THCA tissues significantly decreased compared to normal tissues.


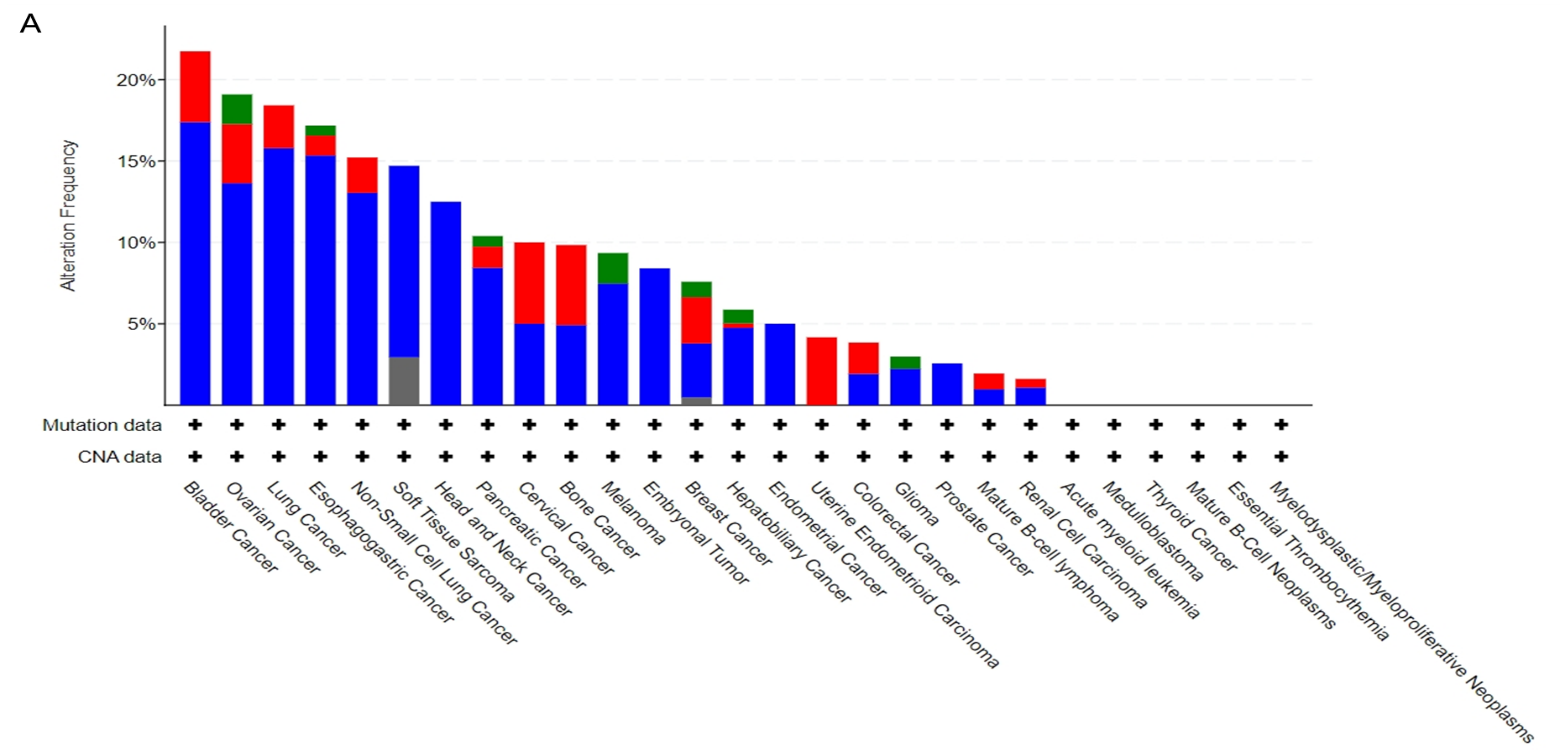


**Figure S5** **Pan-cancer analysis of *UBA1* mutations**

(A) The results show that the highest mutation frequency of *UBA1* in bladder cancer patients is about 27%.


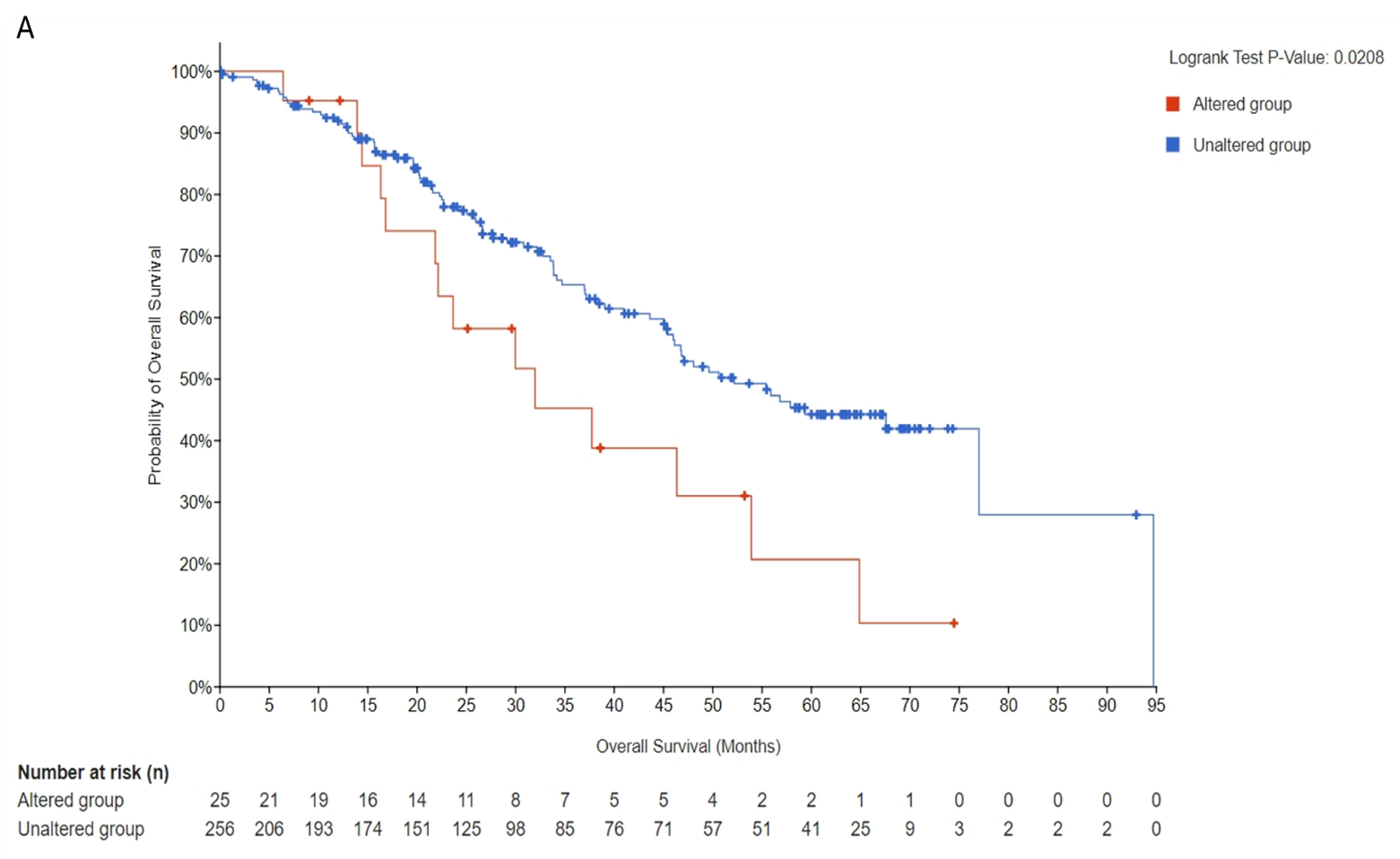


**Figure S6 Analysis of prognostic differences between tumor patients with *UBA1* gene mutations and those without mutations**

(A) Tumor patients with *UBA1* gene mutations have significantly poorer prognosis in OS compared to those without mutations.

**
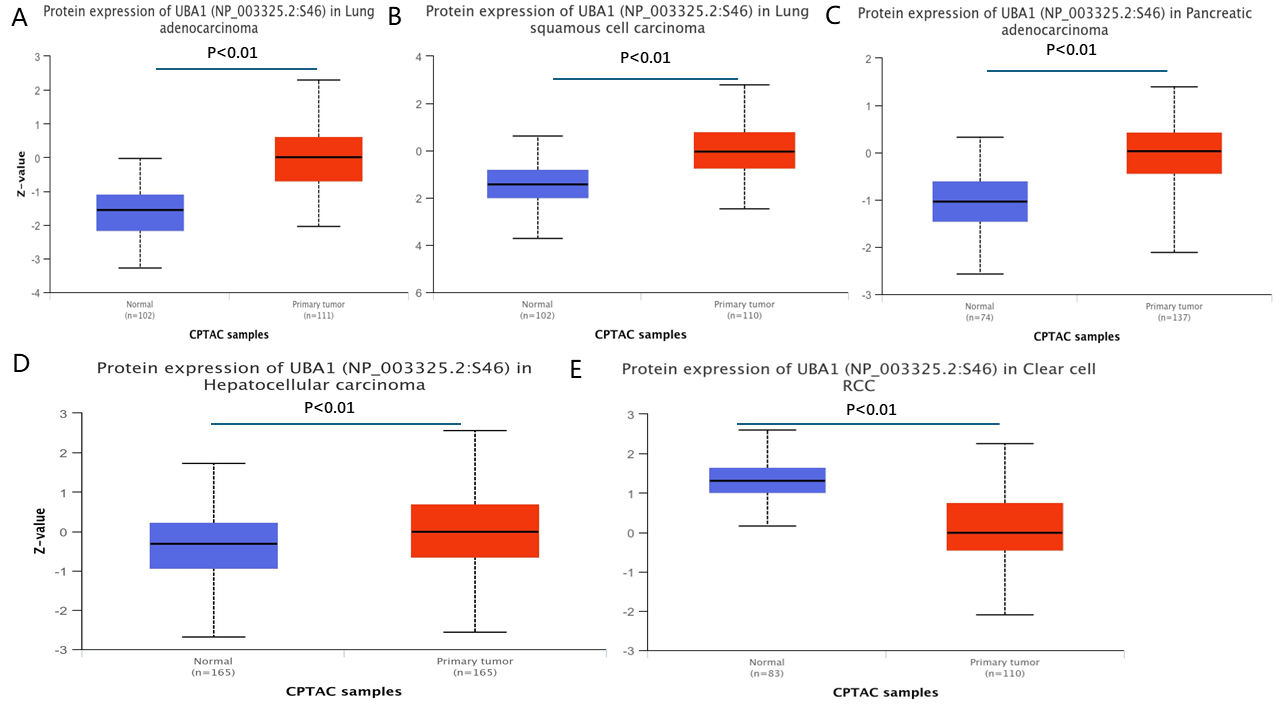
**

**Figure S7 Pan-cancer analysis of *UBA1* phosphorylation**

(A) The CPTAC database analysis found that, compared with normal samples, the S46 phosphorylation of *UBA1* in lung adenocarcinoma increased significantly.

(B) The CPTAC database analysis found that, compared with normal samples, the S46 phosphorylation of *UBA1* in lung squamous cell carcinoma increased significantly.

(C) The CPTAC database analysis found that, compared with normal samples, the S46 phosphorylation of *UBA1* in pancreatic cancer increased significantly.

(D) The CPTAC database analysis found that, compared with normal samples, the S46 phosphorylation of *UBA1* in liver cancer increased significantly.

(E) The CPTAC database analysis found that, compared with normal samples, the S46 phosphorylation of *UBA1* in Clear cell RCC decreased significantly.


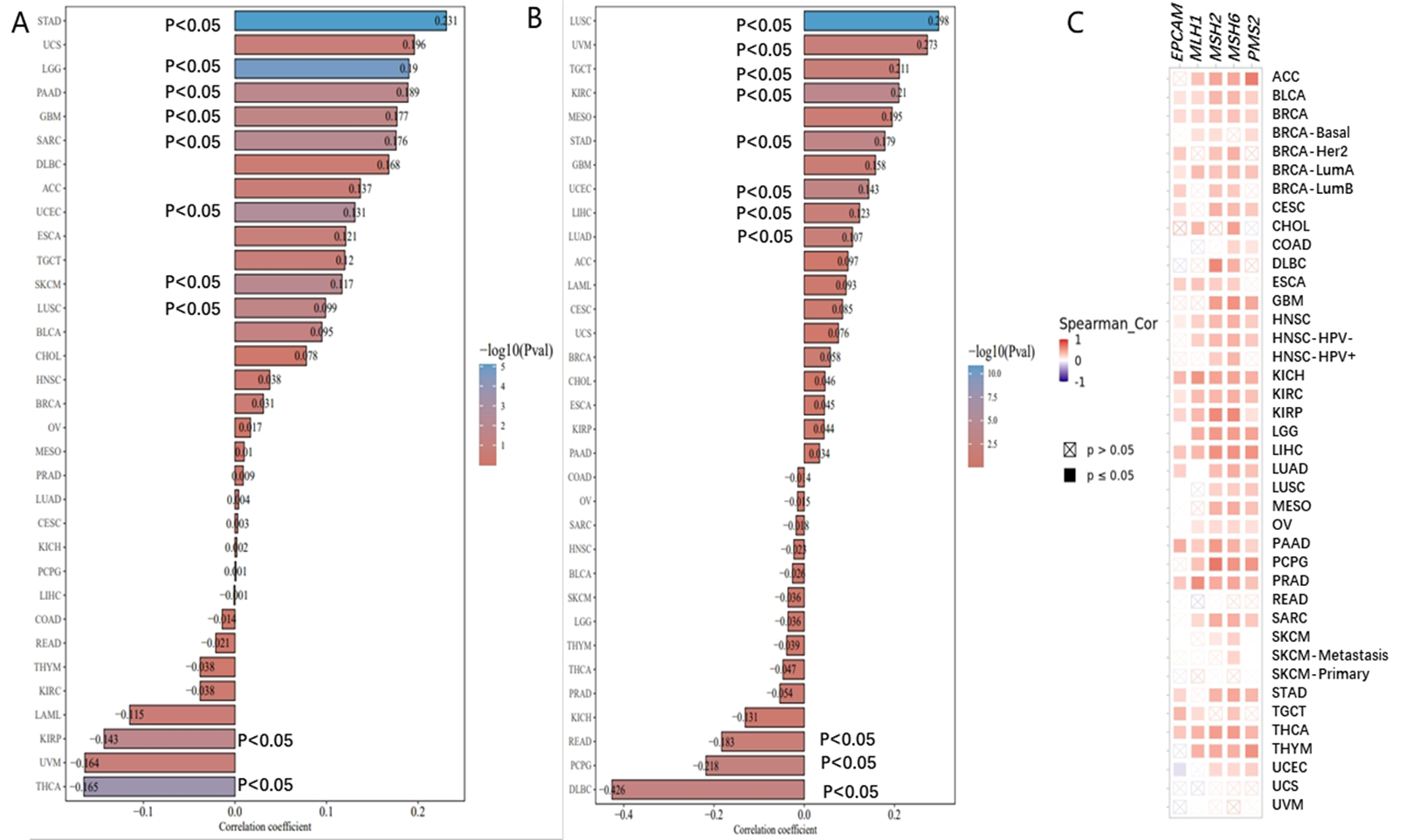


**Figure S8 Pan-cancer analysis of the correlation between *UBA1* expression and immune regulatory factors TMB, MSI and MMR related genes**

(A) *UBA1* expression was significantly correlated with TMB in 10 types of cancer (P<0.05). *UBA1* was positively correlated with TMB in 9 types of tumors including STAD, UCEC, SARC, SKCM, PAAD, GBM, LUSC and LGG, while negatively correlated with TMB in THCA and KIRP.

(B) *UBA1* expression was significantly correlated with MSI in 11 types of cancer (P<0.05). *UBA1* is positively correlated with MSI in 8 types of tumors, including LUSC, KIRC, STAD, UCEC, UVM, TGCT, LUAD and LIHC, while negatively correlated with MSI in DLBC, PCPG and READ.

(C) The correlation between *UBA1* expression and MMR genes was evaluated, including *MLH1, MSH2, MSH6, PMS2* and *EPCAM*. Except for READ, SKCM, uterine carcinosarcoma (UCS)UCS, and UVM, the expression of *UBA1* in most tumors is associated with the expression of the MMR gene.


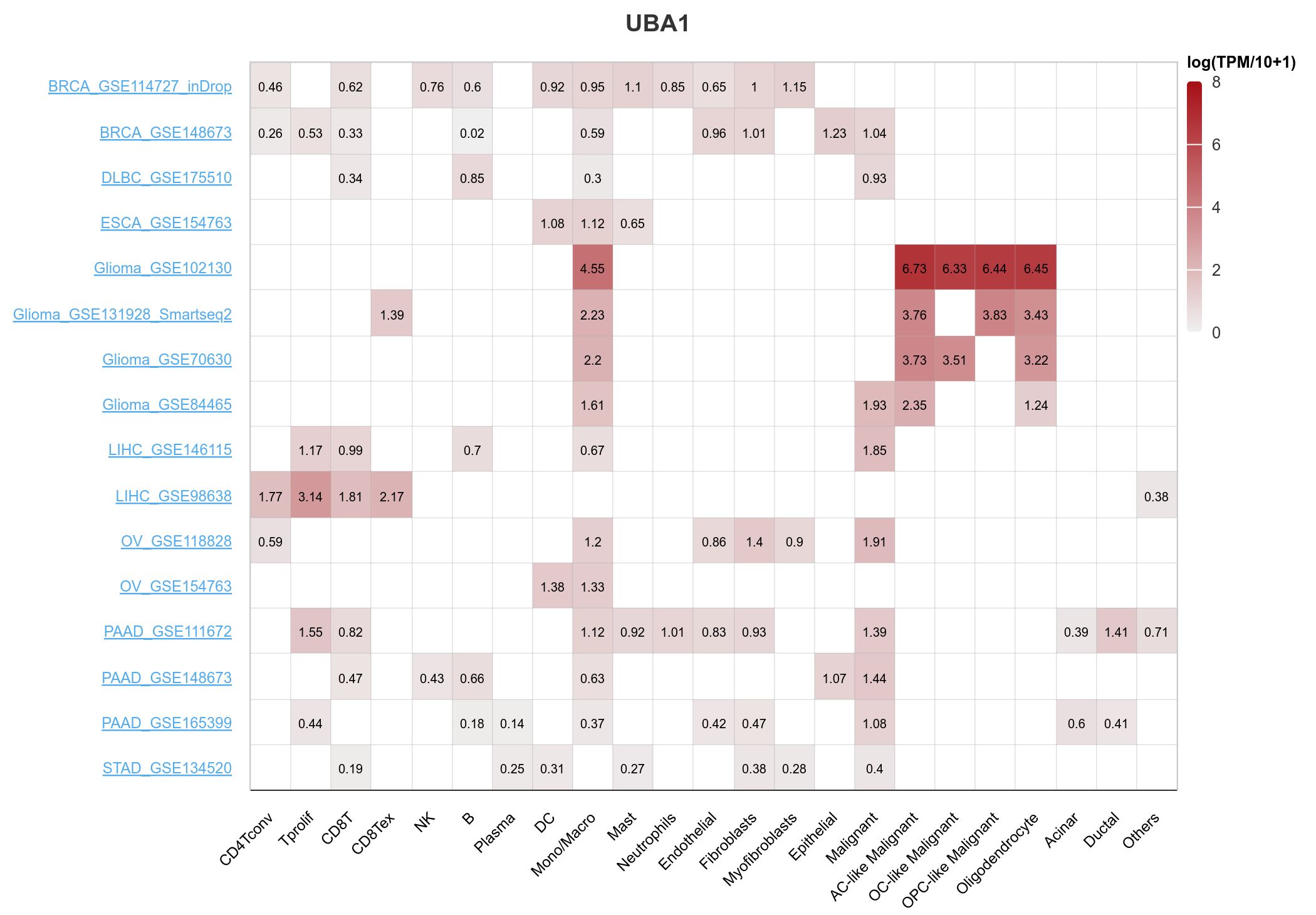


**Figure S9 The expression of *UBA1* on immune cells**

*UBA1* is expressed by macrophages and malignant cells in BRCA, DLBC, glioma, LIHC, OV and PAAD.


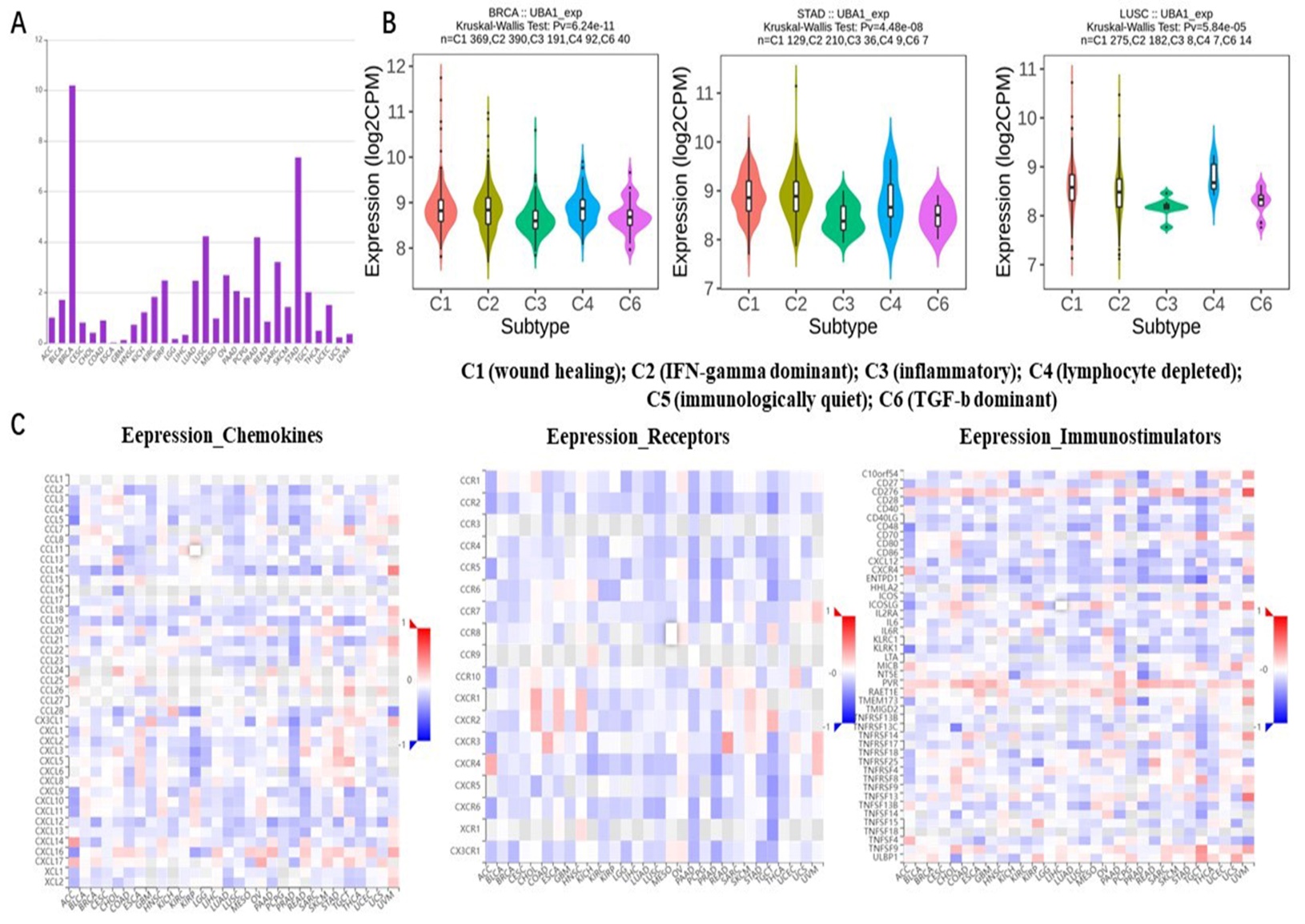


**Figure S10 Analysis of differential expression of *UBA1* in different immune subtypes of cancer based on TISIDB database**

(A) Correlation map between *UBA1* and cancer immune subtypes;

(B) Association diagram between *UBA1* and various subtypes of cancer immunity;

(C) Heat map of the correlation between *UBA1* and chemokines (left), receptors (center) and immune stimulatory molecules (right).


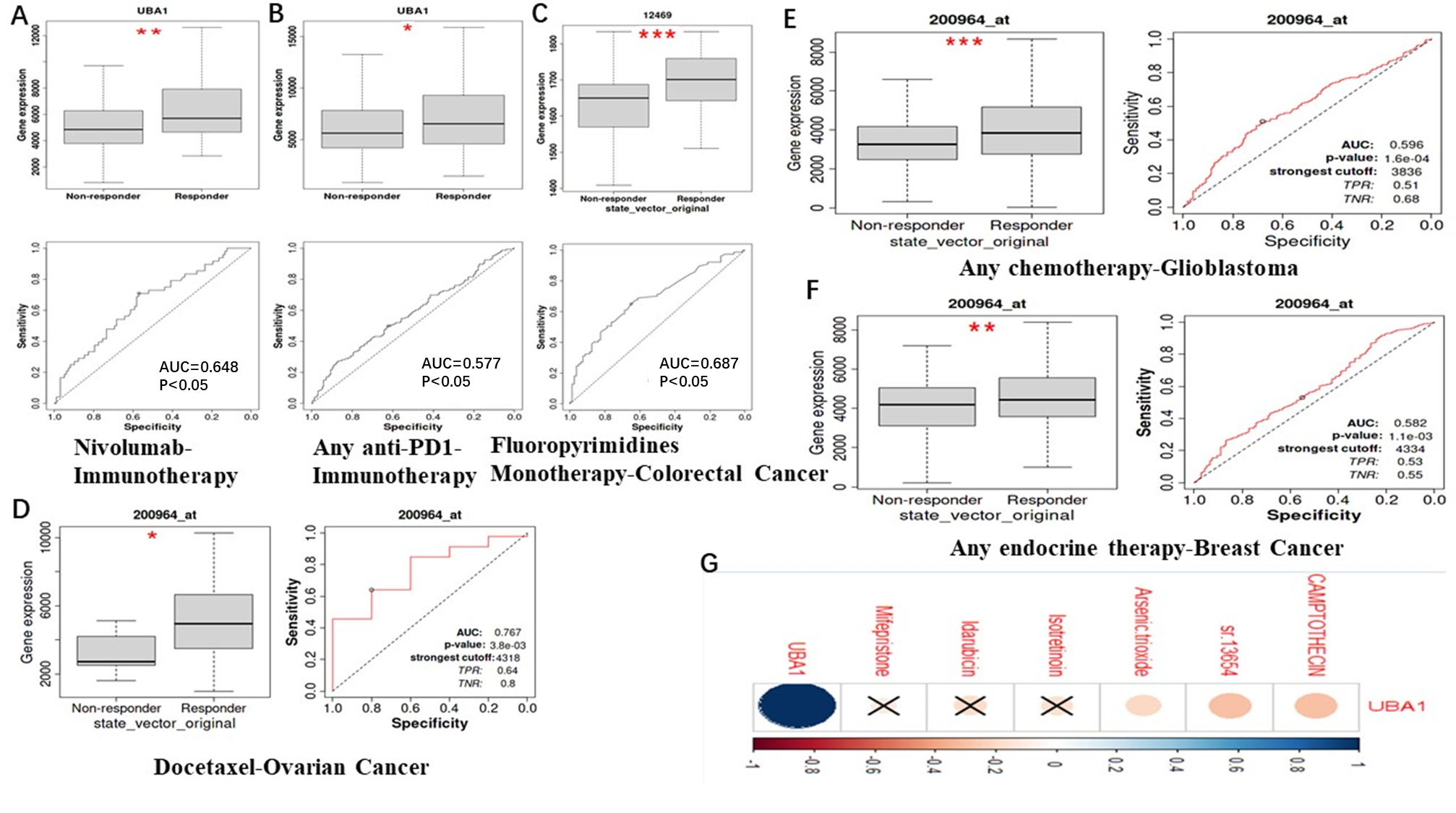


**Figure S11 *UBA1* predicts treatment response to Pan-cancer**

(A) Respondents treated with nivolumab immunotherapy showed higher expression of *UBA1*, with an area under curve (AUC) value of 0.65.

(B) Respondents treated with PD1 immunotherapy showed higher expression of *UBA1*, with an AUC value of 0.58.

(C) In CRC, responders treated with fluoropyrimidine monotherapy showed higher *UBA1* expression, with an AUC value of 0.69.

(D) In OV, responders treated with docetaxel exhibited higher *UBA1* expression, with an AUC value of 0.77.

(E) In GBM, *UBA1* is highly expressed in post chemotherapy responders, with an area under the curve (AUC) value of 0.60.

(F) In BRCA, responders after endocrine therapy had higher *UBA1* expression, with an AUC value of 0.58.

(G) The drug sensitivity of *UBA1* expression in tumors was studied using GSCALite. The expression of *UBA1* is positively correlated with the 50% inhibitory concentration (IC50) values of CAMPTOTHECIN, sr.13654 and Arsenal.


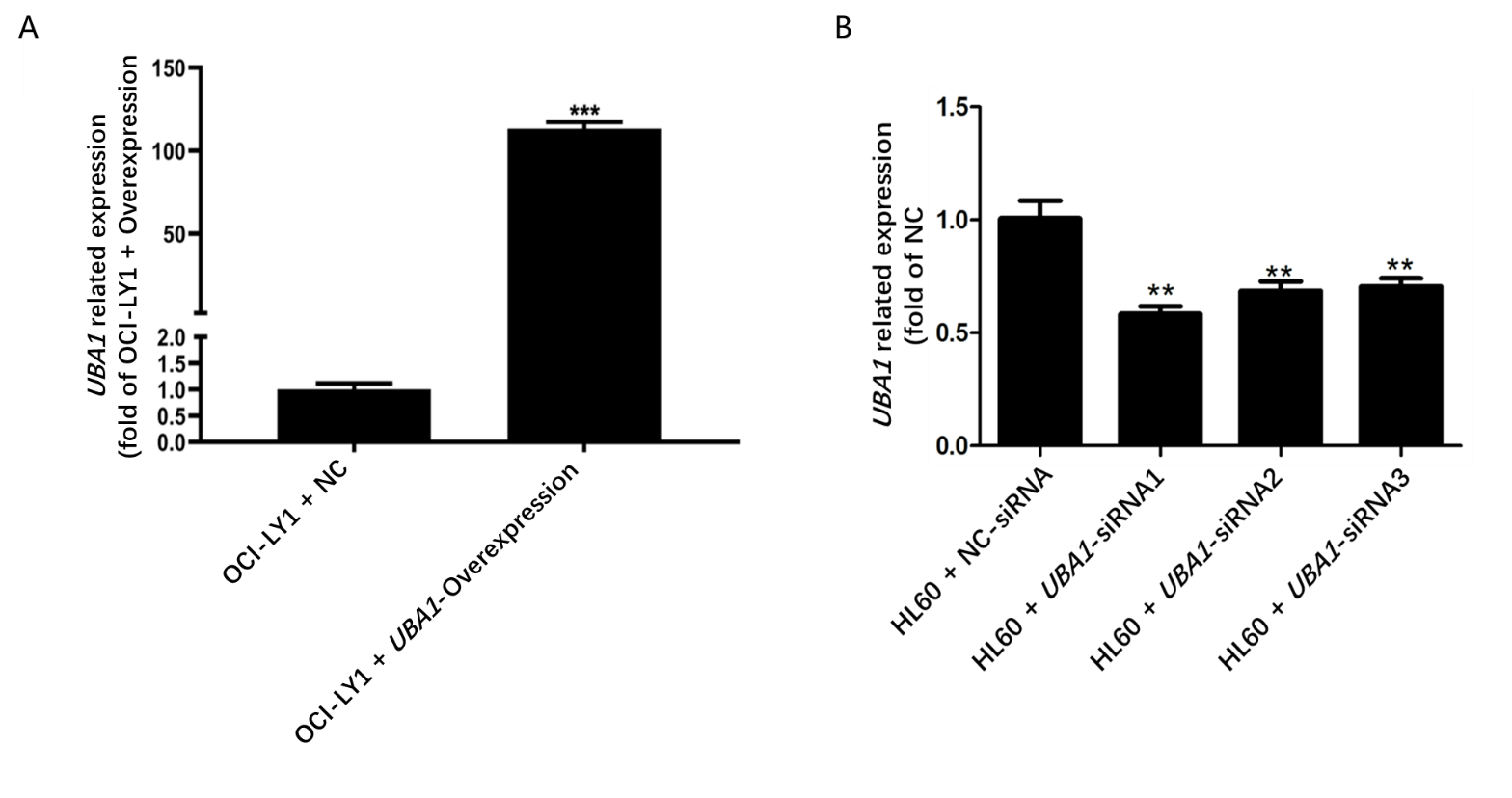


**Figure S12 Construction of *UBA1* overexpression DLBCL cell model and *UBA1* interference AML cell model**

(A) The qPCR results showed that compared with the control group, the cells transfected with OCI-LY1+*UBA1*-Overexpression had the significant overexpression effect on *UBA1* and named OCI-LY1+UBA1-Overexpression.

(B) The qPCR results showed that compared with the control group, the cells transfected with HL-60+*UBA1*-siRNA1 had the most significant interference effect on *UBA1*.
